# *HORMAD1* overexpression predicts response to anthracycline–cyclophosphamide and survival in triple‐negative breast cancers

**DOI:** 10.1002/1878-0261.13412

**Published:** 2023-03-23

**Authors:** Rania El‐Botty, Sophie Vacher, Juliette Mainguené, Adrien Briaux, Sabrina Ibadioune, Ahmed Dahmani, Elodie Montaudon, Fariba Nemati, Léa Huguet, Laura Sourd, Ludivine Morriset, Sophie Château‐Joubert, Thierry Dubois, Virginie Maire, Rosette Lidereau, Audrey Rapinat, David Gentien, Florence Coussy, Ivan Bièche, Elisabetta Marangoni

**Affiliations:** ^1^ Translational Research Department, Institut Curie PSL Research University Paris France; ^2^ Department of Genetics, Institut Curie PSL Research University Paris France; ^3^ Medical Oncology Department, Institut Curie PSL Research University Paris France; ^4^ BioPole Alfort, Ecole Nationale Vétérinaire d'Alfort Maisons Alfort France; ^5^ Faculty of Pharmaceutical and Biological Sciences Paris City University, Inserm U1016 France

**Keywords:** AC sensitivity, biomarker, HORMAD1, TNBC

## Abstract

Triple negative breast cancers (TNBCs) represent 15–20% of all breast cancers and are associated with higher recurrence and distant metastasis rate. Standard of care for early stage TNBC is anthracyclines combined with cyclophosphamide (AC) followed by taxanes, in the neo‐adjuvant or adjuvant setting. This work aimed to identify predictive biomarkers of AC response in patient‐derived xenograft (PDX) models of TNBC and to validate them in the clinical setting. By gene and protein expression analysis of 39 PDX with different responses to AC, we found that high expression of *HORMAD1* was associated with better response to AC. Both gene and protein expression were associated with promoter hypomethylation. In a cohort of 526 breast cancer patients, *HORMAD1* was overexpressed in 71% of TNBC. In a second cohort of 186 TNBC patients treated with AC, *HORMAD1* expression was associated with longer metastasis‐free survival (MFS). In summary, *HORMAD1* overexpression was predictive of an improved response to AC in PDX and is an independent prognostic factor in TNBC patients treated with AC.

## Graphical abstract

Standard of care for early stage TNBC is anthracylines combined with cyclophosphamide (AC) followed by taxanes, in the neo‐adjuvant or adjuvant setting. By gene and protein expression analysis of multiple TNBC PDX we found that high expression of *HORMAD1* was associated with better response to AC *in vivo*. HORMAD1 gene and protein expression was associated with promoter hypomethylation. In a cohort of TNBC patients treated with AC, *HORMAD1* expression was associated with longer metastasis‐free survival (MFS).

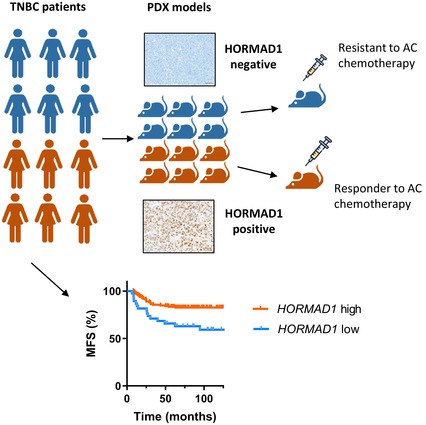

**Abbreviations**
AC: doxorubicin (adriamycine) and cyclophosphamide
BC: breast cancer
BL1: basal‐like 1
BL2: basal‐like 2
ER: estrogen receptor
ERBB2: erb‐b2 receptor tyrosine kinase 2
HBCx: human breast cancer xenograft
HER2: human epidermal growth factor receptor‐2
HR: hazard ratio
HR+: hormone receptor positive
IHC: immunohistochemistry
IM: immunomodulatory
KM: Kaplan–Meier
LAR: luminal‐androgen receptor
LN: lymph node
M: mesenchymal
MFS: metastasis‐free survival
MS‐HRM: methylation‐sensitive high‐resolution melting
MSL: mesenchymal‐stem‐like
OS: overall survival
PD: progressive disease
PDX: patient‐derived xenografts
PR: progesterone receptor
R: regression
RT‐PCR: reverse transcription polymerase chain reaction
RTV: relative tumor volume
SBR: Scarff Bloom Richardson
SD: stable disease
TNBC: triple‐negative breast cancer
UNS: unstable

## 1 Introduction

Triple‐negative breast cancer (TNBC) is defined by a lack of estrogen receptor (ER), progesterone receptor (PR), and of HER2 overexpression. This tumor subtype represents about 15–20% of all breast cancers (BC) [1]. The standard of care for localized TNBC is surgery associated with neo‐adjuvant and/or adjuvant chemotherapy. The recommended regimen is Adriamycin + Cyclophosphamide (AC) followed by paclitaxel [2].

Despite an overall poor prognosis, patients with TNBC patients show a higher response to chemotherapy than patients with other BC types. Nevertheless, patients with TNBC have higher recurrence and distant metastasis rate [3], in particular when response to neo‐adjuvant chemotherapy is incomplete and associated with residual disease at surgery [4]. Several groups have analyzed TNBC heterogeneity with transcriptomic‐based profiling and identified different molecular and biological subtypes, with different sensitivities to neo‐adjuvant therapy [5, 6, 7, 8, 9, 10]. However, these signatures have not been translated to clinical practice.

This highlights the need for identification of predictive biomarkers that can be applied to adapt chemotherapy and improve patients care.

In the present study, we used a cohort of PDX of TNBC to identify predictive markers of response to AC. We found *HORMAD1* among the most up‐regulated genes respectively in AC responder tumors. To validate the preclinical finding in patients' tumors, the prognostic value of *HORMAD1* gene expression, quantified by RT‐PCR analysis, was addressed in a large cohort of TNBC patients treated by chemotherapy, where it was associated with a better survival in univariate and multivariate analysis.

## 2 Materials and methods

### 2.1 Patients' cohorts

Samples of unilateral invasive primary BC excised from patients managed at Institut Curie Hospital (France) from 1978 to 2015 have been analyzed. All patients who entered our institution before 2007 were informed that their tumor samples might be used for scientific purposes and had the opportunity to decline. Since 2007, patients entering our institution have given their approval also by signed informed consent. This study was approved by the local ethics committee (Breast Group of Institut Curie Hospital). The study methodologies conformed to the standards set by the Declaration of Helsinki.

Two cohorts of patients, previously described [11, 12, 13], were analyzed:

- Cohort 1: Samples and clinical data from 526 unilateral invasive primary breast tumors of all molecular subtypes: 101 TNBC, 73 HR^−^ ERBB2^+^, 58 HR^+^ ERBB2^−^ and 294 HR^+^ ERBB2^+^ patients. Mean age at diagnosis was 61 years (range 29–91 years). With a median follow up of 9.2 years (range 1 months to 33 years), 209 patients developed metastasis.
- Cohort 2: Samples and clinical data from 186 patients with unilateral nonmetastatic TNBC treated with AC‐like regimens. Mean age at diagnosis was 53 years (range 28–82 years). 79.7% of patients received anthracycline + cyclophosphamide chemotherapy regimen, 12.4% anthracycline alone and 6.5% other regimens. During a median follow‐up of 7.4 years (range 6 months to 36 years), 43 patients developed distant metastasis.

### 2.2 Patient‐derived xenografts

PDX models were established from primary breast tumors of TNBC patients with informed written consent, in accordance with published protocols [14, 15, 16]. Female Swiss nude mice were purchased from Charles River and maintained under specific pathogen‐free conditions. Their care and housing were in accordance with Institutional Animal Care and French Committee approved criteria (project authorization no. 02163.02). Histology and IHC status (ER, PR, and HER2) was determined for the PDX and compared with that of the patient's initial tumor, as described elsewhere [16, 17].

### 2.3 Assessment of chemosensitivity in PDX

Adriamycin (doxorubicin, Teva Pharmaceuticals) and cyclophosphamide (Endoxan, Baxter) were administered to the mice by the intraperitoneal (i.p.) route at the dose of 2 and 100 mg·kg^−1^, respectively, every 3 weeks [16]. Two cycles of AC treatment were administered and tumor response was evaluated at the end of the second cycle or when tumor volumes reached ethical sizes. For efficacy studies, tumor fragments were transplanted into female 8‐week‐old Swiss nude mice (one fragment was transplanted in each mouse). Xenografts were randomly assigned to the different treatment groups (*n* = 6–10 mice per group) when tumors reached a volume comprised between 100 and 250 mm^3^. Control mice were untreated. Tumor growth was evaluated twice a week by measurement of two perpendicular diameters of tumors with a caliper. Individual tumor volumes were calculated as *V* = *a*x*b* ^2^/2, *a* being the largest diameter and *b* being the smallest. Mice were euthanized when the tumor volume reached the ethical limit of 1500–2000 mm^3^. For each tumor, volumes are expressed relative to the initial volume, as a relative tumor volume (RTV). Percent change in tumor volume was calculated for each tumor as [(*V*_f_ − *V*_0_)/*V*_0_] × 100, where *V*_0_ = initial volume (at the beginning of treatment) and *V*_f_ = final volume (at the end of treatment). Tumor regression (*R*) was defined as a decrease in tumor volume of at least 50%, taking the baseline tumor volume as reference; at least a 35% increase in tumor volume identified progressive disease (PD) and responses that were between +35 and −50% were considered as stable disease (SD) [15].

### 2.4 PDX gene expression: Affymetrix microarray

Transcriptomic profiling of 39 TNBC PDX was performed by gene expression arrays as previously described [14]. GeneChip Human 1.1 ST arrays were hybridized according to Affymetrix recommendations, using the Ambion WT Expression Kit protocol (Life Technologies) and Affymetrix labeling and hybridization kits. Affymetrix CEL files were imported into the Gene Expression Workflow in Partek Genomics Suite version 7.0 (Partek Inc., www.partek.com). Background correction, quantile normalization, log2 transformation, and probeset annotation were performed using default settings for the Robust Multichip Average (RMA) procedure. The molecular subtypes of TNBC PDX models were determined with the web‐based subtyping tool TNBCtype [14, 18]. Given a gene expression data matrix, this tool displays for each tumor sample the predicted subtype, the corresponding correlation coefficient, and the permutation *P*‐value [18].

### 2.5 Plasmid amplification and purification

The plasmids pCMV6‐Myc‐Flag‐HORMAD1 and pCMV6‐Myc‐Flag were purchased from OriGene, USA. Ten nanogram of DNA pCMV6‐Myc‐Flag‐HORMAD1 and pCMV6‐Myc‐Flag were mixed with 25 μL of competent bacteria (Bacteria NEB® 5‐alpha Competent E. coli, #C2987P New England Biolabs) and incubated for 45 min on ice. A heat shock was then performed for 45 s at 42 °C followed by 2 min in ice, before adding 250 μL of SOC media (Invitrogen, 15544‐034) and incubating at 37 °C for 1 h on a shaking incubator. The bacterial transformation mix was plated into a 10 cm LB agar plates containing the appropriate antibiotic and were incubated overnight at 37 °C. The growing colonies were amplified in a preculture of 1.5 mL that was diluted into 400 mL of « ImMedia™ Kan Liquid » media (Invitrogen, #Q61020) and incubated at 37 °C overnight on a shaking incubator. Plasmid DNA was purified using the Macherey‐Nagel (NucleoSpin Plasmid, #740588.50) according to the manufacturer's instruction. Plasmid DNA was then stored at −20 °C.

### 2.6 Cell culture and transfection

The HORMAD1 negative TNBC cell lines BT‐549 (RRID:CVCL_1092) and Hs578T (RRID:CVCL_0332) were purchased from the American Type Culture Collection (ATCC, LGC Promochem, Molsheim, France), authenticated in 2021 by short‐tandem repeat profiling (Powerplex 16 HS, Promega, Charbonnieres les bains, France) and tested for mycoplasma using MycoAlert Mycoplasma Detection Kit (Lonza Biosciences, Durham, NC, USA).

Cell lines were cultured in RPMI1640 glutamax medium (Thermo Fisher Scientific, Courtaboeuf, France, #61870044) supplemented with 10% FBS (Thermo Fisher Scientific, #10270106), 1% penicillin–streptomycin (Thermo Fisher Scientific, #15140122), 1.5 g·L^−1^ sodium bicarbonate (Thermo Fisher Scientific, #25080060) and 10 mm Hepes (Thermo Fisher Scientific, #15630056) and maintained at 37 °C in a humidified atmosphere with 5% CO2.

Twenty‐four hours before plasmid transfection, cells were seeded into six‐well plates (TPP, Trasadingen, Switzerland; #92106) and next day, transfection was performed with 1 μg of plasmid DNA using XTremGene reagent (Sigma, #6366244001) in Opti‐MEM medium (Thermo Fisher Scientific, #31985070), according to the manufacturers' instructions.

### 2.7 Western blot

Proteins were extracted as described previously [19]. Lysates were resolved on 4–12% TGX gels (Bio‐Rad®, Hercules, CA, USA, 4568083), transferred into nitrocellulose membranes (Bio‐Rad®, #170‐4159), and immunoblotted with rabbit antibodies against GAPDH (Cell Signaling Technology, Saint‐Cyr‐L’École, France, #2118) and HORMAD1 (Sigma, # HPA037850). After washes, membranes were incubated with the appropriate secondary antibodies horseradish peroxidase‐conjugated affinity‐purified goat anti‐rabbit (Jackson ImmunoResearch Laboratories, Inc., Ely, UK; Interchim, Clichy, France). Acquisition was performed by Chemidoc MP imager (Bio‐Rad Laboratories).

### 2.8 *In vitro* cell viability assay

Cell viability was assessed with Cell Titer Glo CellTiter‐Glo® Luminescent Cell Viability Assay (Promega; #G7570). Cell viability assays were performed with mycoplasma‐free cells. Cells were plated in 96‐well plates at an appropriate density, in triplicate. They were incubated overnight and transfected with pCMV6‐Myc‐Flag‐HORMAD1 and pCMV6‐Myc‐Flag. After 24 h cells were treated with various concentrations of doxorubicin for 72 h. A volume of CellTiter‐Glo reagent medium was added (v/v). The plates were mixed and incubated 10 min at room temperature. Then luminescence was monitored using Tecan Infinite 200. The effect of doxorubicin concentration and HORMAD1 transfection were analyzed by the two‐way ANOVA test.

### 2.9 RNA extraction and RT‐PCR amplification

Total RNA was extracted from breast specimens by using the acid‐phenol guanidium method as previously described [20]. The quality of the RNA samples was determined by electrophoresis through agarose gels and staining with ethidium bromide, and the 18S and 28S RNA bands were visualized under ultraviolet light. For gene normalization, we used the human TATA box‐binding protein (TBP, GeBbank accession no. NM_003194).

RT‐PCR was performed as described elsewhere [13].

*HORMAD1* gene expression levels were normalized on the basis of *TBP* contents (Genbank accession number NM_003194) used as an endogenous RNA control. The expression values of the tumor samples were subsequently normalized such that the median of the expression values of 13 normal breast tissue samples was one.

mRNA values ≥ 3 were considered as overexpressed. Primers for *HORMAD1* gene were designed with the assistance of oligo 6.0 computer program (National Biosciences, Plymouth, MN, USA). To avoid amplification of contaminating genomic DNA, one of the two primers was placed at the junction between two exons or on two different exons. Agarose gel electrophoresis was used to verify the specificity of PCR amplicons.

### 2.10 HORMAD1 methylation‐sensitive high‐resolution melting (MS‐HRM) analysis.

HORMAD1 promoter methylation was assessed with MS‐HRM analysis that requires DNA pretreatment with sodium bisulfite in order to convert all unmethylated cytosines into uracil. 100 ng of DNA from each sample were treated with sodium bisulfite with the EZ DNA Methylation‐Lightning Kit (Zymo Research, #D5030‐E, Freibourg, Germany), following the manufacturer's instructions. MS‐HRM analysis was performed in a Roche LC480 instrument (Roche Diagnostics, Meylan, France). The promoter regions of HORMAD1 were analyzed using specifically designed primers by Methyldetect (Aalborg, Denmark).

All the analyses were run as follows: 1 cycle of 95 °C for 10 min, 50 cycles of 95 °C for 15 s, 50 °C for 10 s and 72 °C for 15 s; followed by an HRM step of 95 °C for 15 s, 60 °C for 1 min and continuous acquisition to 95°. PCR was performed in a final volume of 20 μL, containing 10 μL LightCycler 480 HRM Master (Roche Diagnostics), 1 μL EpiMelt primer Mix, 2.4 μL MgCl_2_, 0.6 μL H_2_O, and 6 μL of bisulfite modified DNA template. A methylation positive, an assay calibration, and a methylation negative control are supplied with the EpiMelt HORMAD1 Kit (Methyldetect, Aalborg, Denmark). The assay calibration control is included to ensure the assay sensitivity to detect methylation of 1% in each experiment.

### 2.11 Immunohistochemistry

Xenografted tumors were fixed in 10% neutral‐buffered formalin, embedded in paraffin and stained with hematoxylin and eosin. Immunostaining was performed on a Discovery XT Platform (VentanaMedical System, Cambdrige, UK, part of RocheDiagnostics), as previously detailed [15]. The slides were incubated with a rabbit polyclonal antibody against HORMAD1 (SIGMA, #HPA037850). Slides immunostained with rabbit IgG were used as negative controls. Slides were incubated with an anti‐rabbit secondary antibodies (horseradish peroxidase complex) and 3,30‐diaminobenzidine tetrahydrochloride (DAB) as the substrate for color development (ChromoMap Kit with Anti‐rabbit OmniMap, Ventana Medical System). HORMAD1 immunostaining was assessed by determining the intensity and distribution of stained cancer cells. Expression of HORMAD1 was quantified with the H‐score: Sections were scored for intensity (0–3+) and extent (0–100%) of staining. By multiplying intensity and extent of staining, each tumor was assigned an H‐score (range: 0–300). We considered a tumor HORMAD1 negative with H‐score = 0, HORMAD1 low with an H‐score between 1 and 70, and HORMAD1 high when the H‐score was higher than 70, 70 being the median H‐score.

### 2.12 Statistical analysis

### 2.13 PDX studies

For the identification of differentially expressed genes, we used one‐way analysis of variance (ANOVA), log fold changes in expression > 1.5, and *P*‐values < 0.05 to be considered statistically significant. Categorical variables were analyzed with the Fisher's exact test. Two‐tailed unpaired *t*‐tests were used when comparing two groups. Spearman's correlation coefficient was used to assess correlation between mRNA and protein expression. The one‐way ANOVA test was used to analyze the differences of HORMAD1 gene expression in the different TNBC subgroups.

### 2.14 Patient cohorts

The histological and clinical data were compared in χ^2^ or Fisher's exact tests, as appropriate. Metastasis‐free survival (MFS) was defined as the time interval from the diagnosis to the date of first evidence of distant metastases confirmed by imaging or histologic evidence. Survivals were estimated by the Kaplan–Meier method, and survival curves were compared in log‐rank tests. Subjects were censored at the date of last follow up. Multivariate analysis using a Cox proportional hazards model assessed the predictive value for MFS and OS of the parameters with a *P*‐value < 0.1 on univariate analysis, including lymph node (LN) status, macroscopic tumor size, molecular histology, HORMAD1 mRNA expression. For all statistical tests, the limit of significance was defined as *P* < 0.05. Analyses were performed using prism 7.0 software (Graph Pad Inc., Boston, MA, USA).

## 3 Results

### 3.1 Hormad1 expression is associated with response to AC in PDX of TNBC

**Fig. 1:**
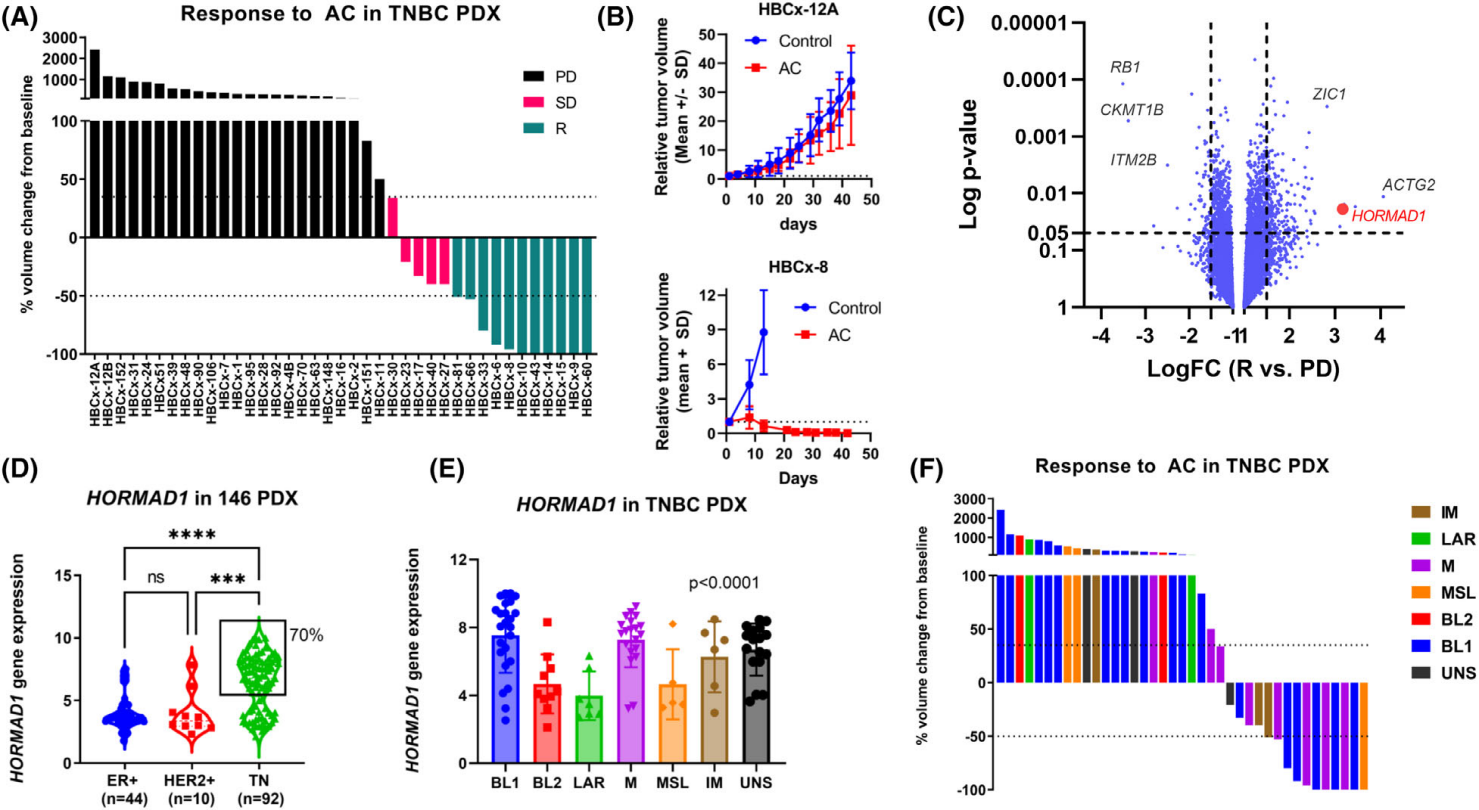
Chemotherapy response and HORMAD1 expression in TNBC PDX. (A) Waterfall plot representing response to AC (doxorubicin and cyclophosphamide) in 39 TNBC PDX. Each bar represents the median change in tumor volume from baseline in treated xenografts. PD, progressive disease; R, regression (response); SD, stable disease. (B) *In vivo* response to AC in 2 PDX models (HBCx‐8 and HBCx‐12A). Mean ± SD. *n* = 8 mice/group for the HBCx‐8 and *n* = 10 mice/group for the HBCx‐12A. *In vivo* experiments were not replicated. (C) Volcano plot representing gene expression changes between responding (R) and resistant PDX (PD). FC: fold change (D) *HORMAD1* expression in 146 PDX established from ER+, HER2+ and TN breast cancers (transcriptomic gene expression). ****P < 0.0001 ***P < 0.001, Tukey's multiple comparisons test (one‐way ANOVA). (E) *HORMAD1* expression in the different molecular subtypes of TNBC (*n* = 66 TNBC PDX). BL1, basal‐like 1; BL2, basal‐like 2; IM, immunomodulatory; LAR, luminal androgen receptor; M, mesenchymal; MSL, mesenchymal‐stem like; UNS, unstable. Mean ± SD (one‐way ANOVA). (F) Response to AC in PDX models of different TNBC subtypes.

We evaluated the antitumor activity of the standard of care AC regimen in 39 PDX of TNBC (Fig. 1A, Figs S1 and S2). Among the 39 PDX, 11 (28%) achieved complete or partial response (R), 23 (59%) had tumor progression (PD), and 5 (13%) showed stable disease (SD). The response to AC of 2 PDX models is shown as example in Fig. 1B. A differential expression analysis of transcriptomic datasets comparing R versus PD PDX identified 271 differentially expressed genes (Table S1). *HORMAD1* gene was among the top up‐regulated genes (Fig. 1C).

*HORMAD1* is meiosis‐associated gene, whose expression has been described in a subset of TNBC and is associated with response to cisplatin [21]. However, data on HORMAD1 expression in BC remain scarce. To have an overview of *HORMAD1* expression in the whole cohort of our PDX (*n* = 146), we analyzed its transcriptomic expression in the different subsets of BC. We found that *HORMAD1* expression was higher in TNBC as compared to ER^+^ and HER2^+^ PDX (*P* < 0.0001 and *P* = 0.0008, respectively) and that it presented a bimodal expression with 70% of samples showing high transcriptomic expression (Fig. 1D). We next asked whether *HORMAD1* expression was higher in specific molecular subtypes of TNBC, classified according to the Lehman classification [8, 14]. We found that its expression was higher in BL1, IM and M subgroups, while it was lower in MSL, BL2, and LAR subtypes (*P* < 0.0001, ANOVA test; Fig. 1E) (Table S2). Accordingly, AC‐responding PDX models were mainly of BL1, M, and IM subtypes (Fig. 1F).

**Fig. 2:**
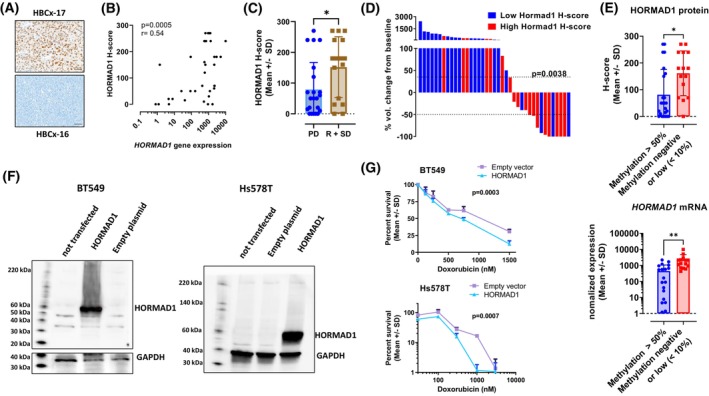
HORMAD1 protein expression in PDX. (A) Immunohistochemistry analysis of HORMAD1 expression in HBCx‐17 and HBCx‐16 PDX models (scale 50 μm). The images shown are representative of the whole tissue sections. The same representative samples that are also shown in Fig. S3. (B) correlation between HORMAD1 gene and protein expression (H‐score) in the 39 TNBC PDX (spearman). (C) HORMAD1 H‐score in PDX models responding to AC (R and SD groups) as compared to PDX models not responding (PD group). Mean ± SD, *P < 0.05 (unpaired *t*‐test). (D) Response to AC in PDX models according to HORMAD1 expression. High and low H‐scores were defined as > and < than 70 (70 = median H‐score in the PDX cohort). Fisher's exact test. (E) HORMAD1 gene and protein expression according to *HORMAD1* promoter methylation. Mean ± SD, *P < 0.05; ** < 0.01 (unpaired *t*‐test). (F) Western blot analysis of HORMAD1 and GAPDH protein in the BT549 and Hs578T cell lines transfected with the plasmid containing the HORMAD1‐cDNA or the empty plasmid. Western blots were not replicated. (G) BT549 and Hs578T cell viability in the presence of different concentrations of doxorubicin. Cell viability assays were replicated twice, and the results were analyzed by the two‐way ANOVA test.

HORMAD1 protein, analyzed by immunohistochemistry, was detectable in 32/39 TNBC PDX (82%), with heterogeneous expression level (Fig. S3). Figure 2A shows as examples a PDX showing a strong expression of HORMAD1 (HBCx‐17) and one HORMAD1 negative (HBCx‐16). HORMAD1 protein and gene expression, quantified by the H‐score and by RT‐PCR analysis, respectively, were significantly correlated (Fig. 2B). HORMAD1 protein expression, was higher in the group of tumors responding to AC (regression + stable disease groups) as compared to PDX models in the “progressive disease” (PD) subgroup (Fig. 2C). We next classified TNBC PDX models over‐expressing or underexpressing HORMAD1 based on the median value of the H‐score (Fig. 2D). High HORMAD1 expression was significantly associated with response to AC (including R and SD) (*P* = 0.0038, Fischer's exact test).

We then analyzed the percentage of *HORMAD1* promoter methylation and found that it was higher than 50% for 25 PDX (64%), 10% in 3 PDX (8%), < 1% or 0% for 14 PDX (36%). HORMAD1 gene and protein expression were lower in PDX with more than 50% of promoter methylation (Fig. 2E).

Finally, we analyzed the response to doxorubicin in two HORMAD1 negative TNBC cell lines (BT‐549 and Hs578T) that were transfected with a plasmid containing the *HORMAD1* cDNA. Figure 2F shows the expression of HORMAD1 protein, assessed by western blot analysis, after transfection with an empty plasmid (PCMV6) and with a HORMAD1‐cDNA plasmid in BT‐549 and Hs578T cells. HORMAD1 overexpression decreased cell viability of BT549 and Hs578T cells treated by doxorubicin (Fig. 2G; *P* = 0.0003 and *P* = 0.0007, respectively, two‐way ANOVA test). We also exposed HORMAD1‐transfected Hs578T cells to different concentrations of 4‐hydroxycyclophosphamide, the active metabolite of cyclophosphamide, without finding any difference in cell sensitivity (data not shown).

### 3.2 Expression of HORMAD1 is prognostic in TNBC patients

To validate the preclinical findings at the clinical level, we analyzed *HORMAD1* expression by RT‐PCR analyses in two cohorts of BC.

**Fig. 3:**
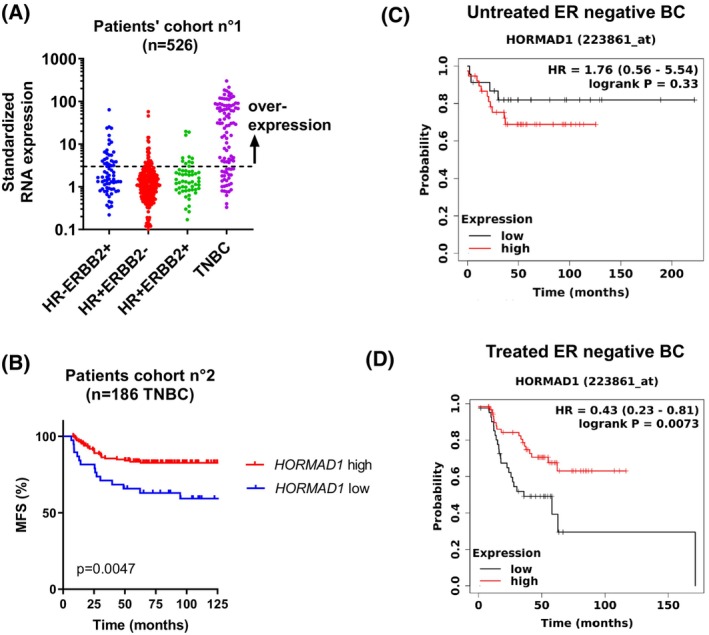
*HORMAD1* expression in different cohorts of breast cancer. (A) HORMAD1 gene expression in patients' cohort n°1 (*n* = 526 breast cancer samples), determined by RT‐PCR analysis. Expression values of the tumor samples were normalized on the median expression value of 13 normal breast tissue samples. mRNA values ≥ 3 were considered as overexpressed. *N* = 73 patients in the RH^−^ ERBB2^+^ group, *n* = 294 in the RH^+^ ERBB2^+^ group, *n* = 58 in the RH^+^ ERBB2^+^ group and *n* = 101 in the TNBC group. (B) Metastasis‐free survival (MFS) of the cohort n°2, including 186 TNBC, according to *HORMAD1* evaluated by RT‐qPCR. *HORMAD1* was classified as high or low based on the cut‐off of 3.4. *P*‐values are calculated with the log‐rank analysis. (C) Survival analysis from the Kaplan–Meier Plotter of untreated (*n* = 61) ER‐negative breast cancer patients according to high or low expression of *HORMAD1* (optimal cut‐off) HR = hazard ratio. (D) Survival analysis from the Kaplan–Meier Plotter of treated (*n* = 103) ER‐negative breast cancer patients according to high or low expression of *HORMAD1* (optimal cut‐off). HR, hazard ratio.

We first evaluated HORMAD1 mRNA expression in the cohort 1 consisting of 526 patients with BC of all molecular subtypes. Patients' characteristics, treatment, and key prognostic factors are represented in Table S3. *HORMAD1* was overexpressed in 71% of TNBC, 36% of the HR^−^ ERBB2^+^ group, 12% of the HR^+^ ERBB2^−^ group and 19% of the HR^+^ ERBB2^+^ group (*P* < 0.0001) Fig. 3A.

Next, we analyzed *HORMAD1* expression in a second cohort of 186 patients treated for unilateral nonmetastatic TNBC, described in Table S4. During a median follow‐up of 7.4 years, 43 patients developed distant metastasis (23%). *HORMAD1* was overexpressed in 80% of our samples and HORMAD1 overexpression was associated with high SBR grade (Table 1). In this second cohort, metastasis‐free survival (MFS) was longer in patients with *HORMAD1* overexpression (*P =* 0.014; Fig. 3B). Multivariate analysis using a Cox proportional hazards model assessed the predictive value for MFS and the parameters with a *P*‐value < 0.1 in univariate analysis, including LN status, macroscopic tumor size and *HORMAD1* mRNA expression (Table S5). The prognostic significance of LN status (*P* = 0.0001), macroscopic tumor size (*P* = 0.029) and *HORMAD1* mRNA expression persisted in multivariate analysis.

**Table 1:** Association between HORMAD1 mRNA expression and histopathological and clinical characteristics of 186 triple negative breast cancer patients. NS, not significant.

	Number of patients (%)	HORMAD1 low	HORMAD1 high	*P*‐value^a^
Total	186 (100.0)	37 (19.9)	149 (80.1)	
Age				
≤ 50	78 (41.9)	14 (17.9)	64 (82.1)	0.57 (NS)
> 50	108 (58.1)	23 (21.3)	85 (78.7)	
SBR histological grade^b^				
II	15 (8.1)	7 (46.7)	8 (53.3)	**0.014**
III	171 (91.9)	30 (17.5)	141 (82.5)	
Lymph node status^c^				
Negative	109 (58.9)	18 (16.5)	91 (83.5)	0.16 (NS)
Positive	76 (41.1)	19 (25.0)	57 (75.0)	
Macroscopic tumor size				
≤ 25 mm	112 (60.2)	19 (17.0)	93 (83.0)	0.22 (NS)
> 25 mm	74 (39.8)	18 (24.3)	56 (75.7)	
Radiotherapy^c^				
Yes	168 (90.8)	33 (19.6)	135 (80.4)	0.90 (NS)
No	17 (9.2)	3 (17.6)	14 (82.4)	
Chemotherapy regimen^d^				
AC	122 (79.7)	26 (21.3)	96 (78.7)	0.14 (NS)
Anthracyclines alone	19 (12.4)	0 (0.0)	19 (100)	
Other	10 (6.5)	2 (20.0)	8 (80.0)	
No chemotherapy	2 (1.3)	0 (0.0)	2 (100)	

a Chi‐square test.

b Scarff Bloom Richardson classification.

c Information available for 185 patients.

d Information available for 153 patients.

Two unrelated molecular markers may provide a more accurate prediction of prognostic when combined than when considered separately. *HORMAD1* status was not related to macroscopic tumor size and LN status in this series. By combining *HORMAD1* status and macroscopic tumor size status, we identified four separate prognostic groups with significantly different MFS curves (*P* = 0.0056; Fig. S4A). The patients with the poorest prognosis had low *HORMAD1* expression and large tumor size (> 25 mn), while those with the best prognosis had high *HORMAD1* expression and small tumor size (≤ 25 mn).

By combining *HORMAD1* expression and LN status, we identified three separate prognostic groups with significantly different MFS curves (*P* < 0.0001; Fig. S4B). The patients with the poorest prognosis had low *HORMAD1* expression and positive LN while those with the best prognosis had negative LN (whatever the level of *HORMAD1* expression).

Finally, we analyzed its expression by the Kaplan–Meier plotter in ER negative BC patients treated or not by chemotherapy [22]. In untreated patients (*n* = 61), *HORMAD1* expression was not associated with survival (HR = 1.76, *P* = 0.33) (Fig. 3C), while in treated patients (*n* = 103), high *HORMAD1* gene expression was associated with a better survival (HR = 0.43, *P* = 0.0073; Fig. 3D).

## 4 Discussion

HORMAD1 belongs to a family of proteins characterized by a HORMA domain that is present in several DNA repair and cell cycle factors [23, 24]. In healthy adults, its expression is restricted to male germ cells, but re‐expression can be observed in cancer [25, 26]. HORMAD1 aberrant expression is reported in lung cancers [27], TNBC [21, 28], ovarian cancers [29], and gastric cancers [30]. HORMAD1's precise role and prognostic value in cancer patients is controversial.

The bimodal pattern of *HORMAD1* expression observed in TNBC was previously reported in different types of cancer, including BC [31]. The mechanism, by which HORMAD1 re‐expression occurs, is promoter demethylation, frequently associated with re‐emergence of cancer testis antigen [25, 31, 32, 33]. Accordingly, the analysis of promoter methylation in our set of PDX models showed a lower percentage of *HORMAD1* methylation in PDX with high expression of HORMAD1 gene and protein.

Published data in the predictive value of HORMAD1 in BC patients are rare. Chen et al. [34] reported HORMAD1 overexpression to be associated with worse outcomes in a cohort of 240 TNBC patients, while in our study, high *HORMAD1* expression is associated with longer MFS. A possible explanation of this discrepancy could be that our cohort is exclusively composed of patients treated by chemotherapy. This hypothesis is also supported by survival data from the KM plotter showing that high HORMAD1 expression is associated with a better survival only in ER negative BC patients treated by chemotherapy.

The finding that HORMAD1 expression is associated with response to chemotherapy could be related to its function in DNA repair. Indeed, it has been shown by Watkins et al. [21] that HORMAD1 expression suppresses homologous recombination and double‐strand break repair, leading to increased sensibility to cisplatin and PARP inhibitors. The same group recently reported that tumor cells expressing HORMAD1 have specific vulnerabilities related to their ability to repair DNA damage or replicate through damaged DNA [35]. However, in our cell lines, the sensitivity to doxorubicin was increased only at the highest drug concentration and the difference was modest, therefore, we cannot conclude that HORMAD1 is driving doxorubicin response.

An opposite function of HORMAD1 was described in lung cancer, where HORMAD1 has been shown to promote DNA damage repair in response to ionizing radiation and camptothecin, and in TNBC cell lines treated by docetaxel, where HORMAD1 knockdown enhanced apoptosis [36]. This suggests that the function of HORMAD1 in the treatment response may diverge in different cancers models and may depend on the type of chemotherapy [37]. Other factors such as culture conditions, cell cycle state, or dominant‐negative effects of the ectopically expressed HORMAD1 have been raised to explain discrepancies between the different studies [37].

## 5 Conclusions

In conclusion, our work provides evidence that high expression of HORMAD1 is associated with better response to chemotherapy in TNBC PDX and patients. Further work will be necessary to determine the role of HORMAD1 in driving chemotherapy response in TNBC.

## Conflict of interest

The authors declare no conflict of interest.

## Author contributions

JM, SV, RE‐B, AB, SI, and RL were involved in acquisition and analysis of RT‐PCR data and analysis of HORMAD1 promoter methylation. AD, EMo, FN, LH, LS, and LM established the PDX models and performed *in vivo* experiments. SC‐J was involved in acquisition and analysis of IHC data. TD, VM, AR, and DG performed the transcriptomic analyses. FC updated the clinical data of the patients cohorts. EMa and IB were involved in the study design and wrote the paper.

## Supporting information


**Fig. S1.** Response to AC chemotherapy in TNBC PDX (from HBCx‐1 to HBCx‐30). Adriamycin and cyclophosphamide were administered to the mice by the intraperitoneal (i.p.) route at the dose of 2 and 100mg/kg respectively, every 3 weeks Two cycles of AC treatment were administered, and tumour response were evaluated at the end of the second cycle or when tumour volumes reached ethical sizes. Volumes are expressed relative to the initial volume, as a relative tumour volume (RTV). Mean +/‐SD, n=6‐8 mice/group.

**Fig. S2.** Response to AC chemotherapy in TNBC PDX (from HBCx‐31 to HBCx‐162). Adriamycin and cyclophosphamide were administered to the mice by the intraperitoneal (i.p.) route at the dose of 2 and 100mg/kg respectively, every 3 weeks Two cycles of AC treatment were administered and tumour response were evaluated at the end of the second cycle or when tumour volumes reached ethical sizes. Volumes are expressed relative to the initial volume, as a relative tumour volume (RTV). Mean +/‐SD, n=6‐8 mice/group.

**Fig. S3.** Immunohistochemistry analysis of HORMAD1 in the 39 PDX. Images are representative of the whole tissue sections; scale bar is 50 μm.

**Fig. S4.** (A) MFS survival curves of TNBC patients stratified according to *HORMAD1* status and macroscopic tumour size status (T). The patients with the poorest prognosis had low *HORMAD1* expression and large tumour size (> 25 mn), while those with the best prognosis had high *HORMAD1* expression and small tumour size (≤25mn). (B) MFS survival curves of TNBC patients stratified according to *HORMAD1* status and LN status. The patients with the poorest prognosis had low *HORMAD1* expression and positive LN while those with the best prognosis had negative LN (whatever the level of *HORMAD1* expression).

**Table S1.** List of genes differentially expressed between PDXs treated by AC in the “response group (R)” as compared to “progressive disease (PD)” group.

**Table S2.** statistical analysis of Fig. 1E (*HORMAD1* expression in the different subgroups of TNBC).

**Table S3.** Histopathological and clinical characteristics of 526 breast cancer patients. ^a^Log‐rank test (521 samples with MFS > 6 months). NS: not significant; ^b^Scarff Bloom Richardson classification, ^c^Information available for 511 patients; ^d^Information available for 521 patients; ^e^Information available for 516 patients.

**Table S4.** Histopathological and clinical characteristics of 186 triple negative breast cancer patients. NS: not significant, AC: anthracyclines cyclophosphamide; ^a^Log‐rank test, ^b^Scarff Bloom Richardson classification, ^c^Information available for 186 patients; ^d^Information available for 153 patients.

**Table S5.** Multivariate COX analysis of MFS for HORMAD1 in the series of 186 triple negative breast cancers. ^a^Hazard ratio. ^b^95% Confidence Interval. ^c^Multivariate COX analysis.

## Data Availability

The gene expression data that support the findings of this study are available on request from the corresponding author. The data are not publicly available due to privacy or ethical restrictions.
