# Supplementary figures and images for: HORMAD1 overexpression predicts response to anthracycline–cyclophosphamide and survival in triple‐negative breast cancers
Source: Mol Oncol. 2023 Mar 23;17(10):2017–28. doi: 10.1002/1878-0261.13412 (PMC10552896; doi:10.1002/1878-0261.13412)

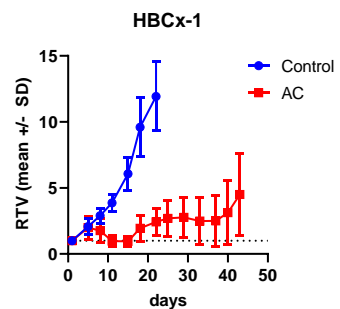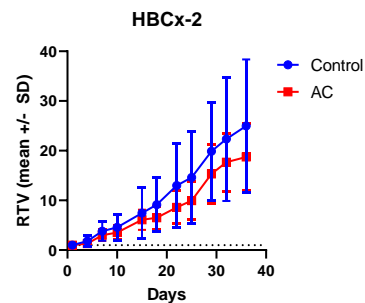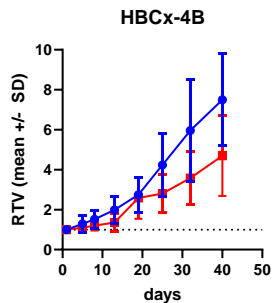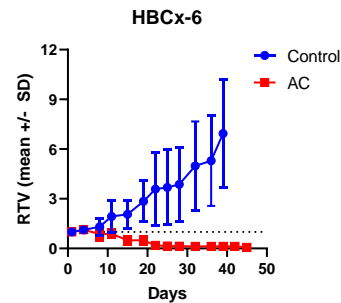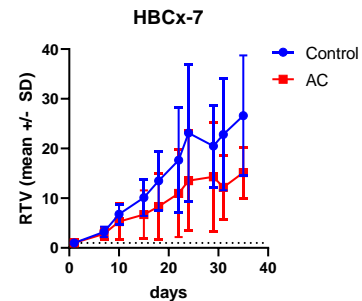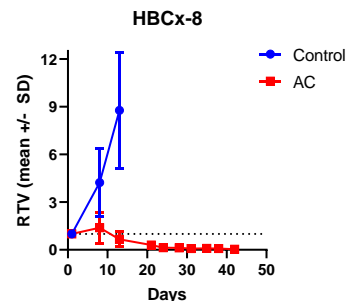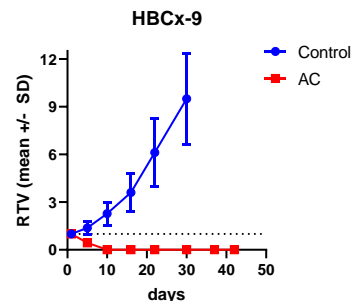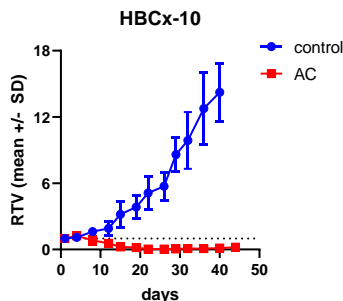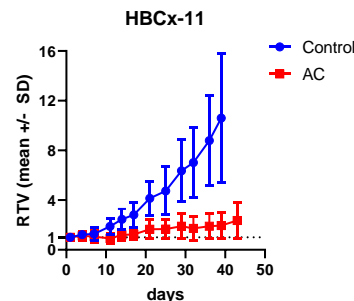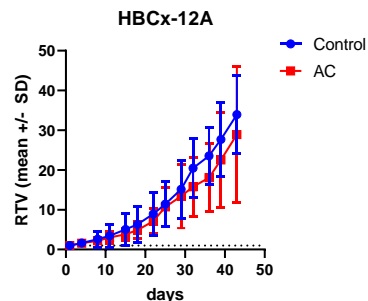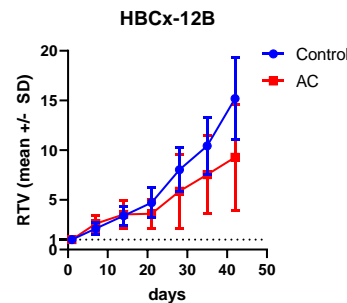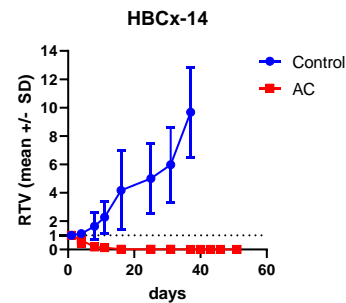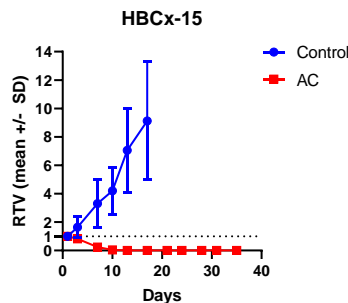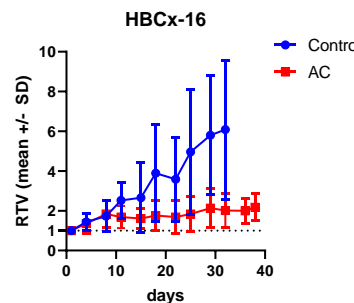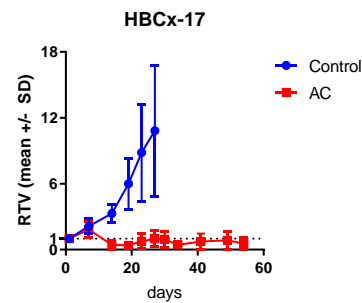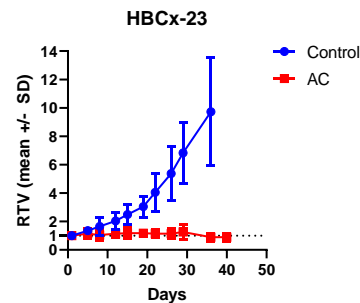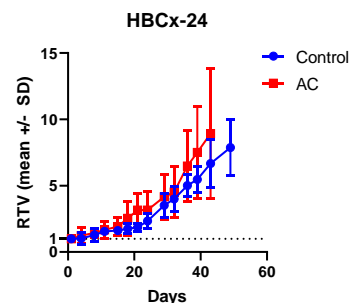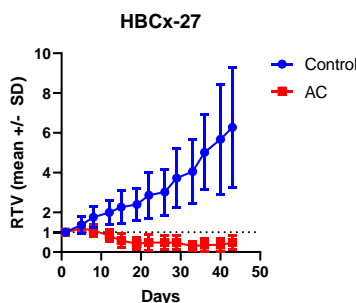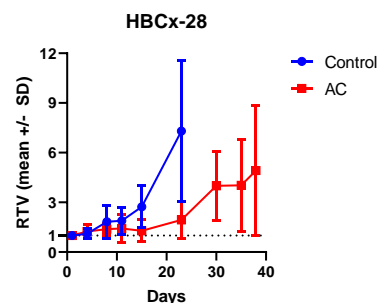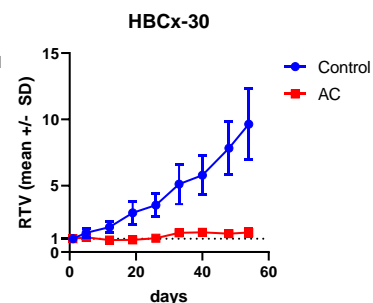

Supplement: Supplementary file 1 — Fig. S1. Response to AC chemotherapy in TNBC PDX (from HBCx‐1 to HBCx‐30). Adriamycin and cyclophosphamide were administered to the mice by the intraperitoneal (i.p.) route at the dose of 2 and 100mg/kg respectively, every 3 weeks Two cycles of AC treatment were administered, and tumour response were evaluated at the end of the second cycle or when tumour volumes reached ethical sizes. Volumes are expressed relative to the initial volume, as a relative tumour volume (RTV). Mean +/‐SD, n=6‐8 mice/group. [file MOL2-17-2017-s002.pdf]

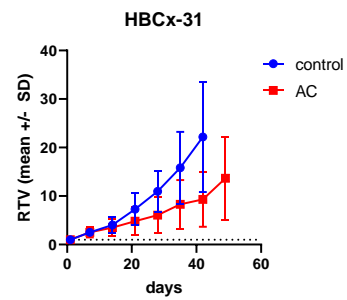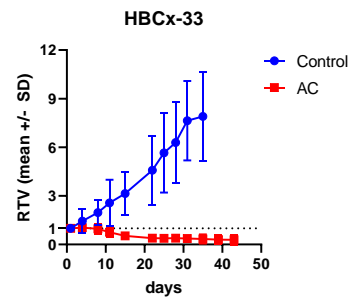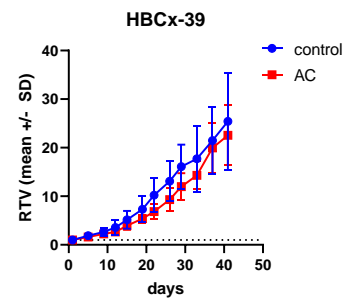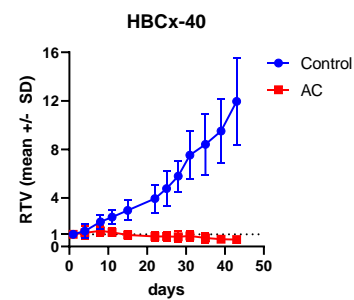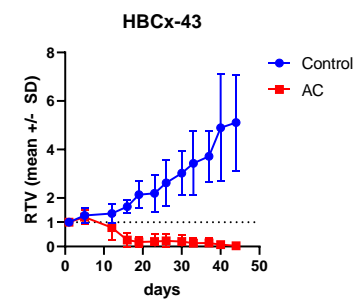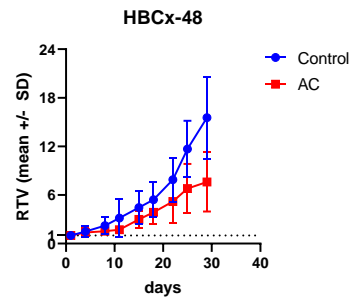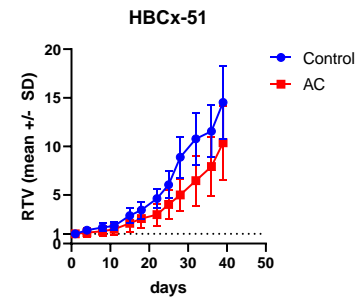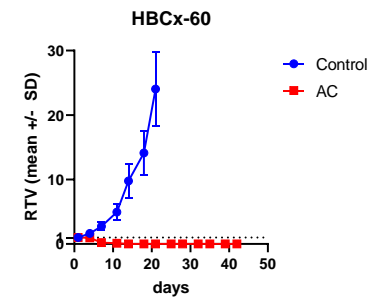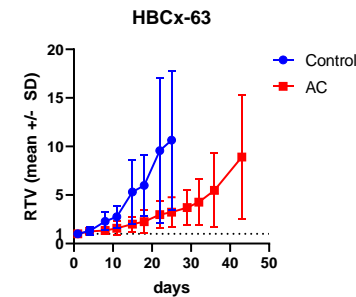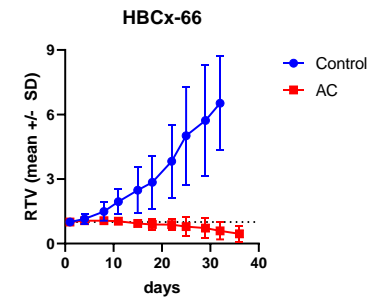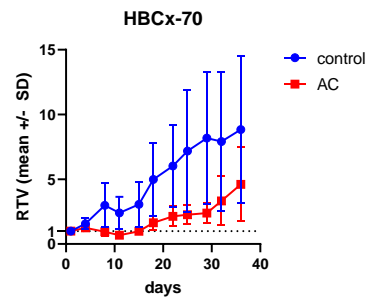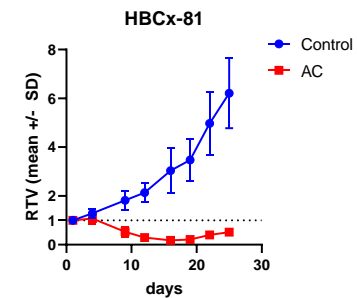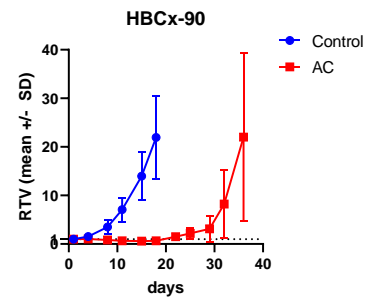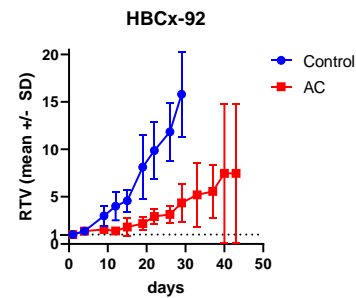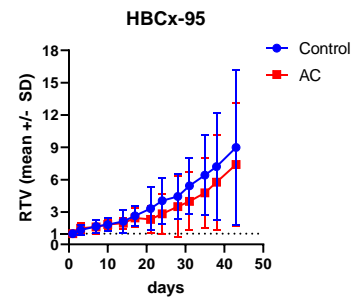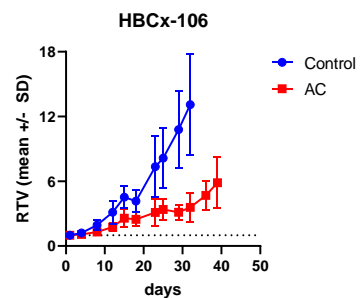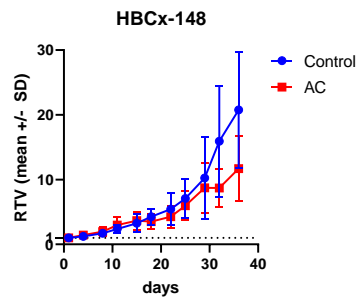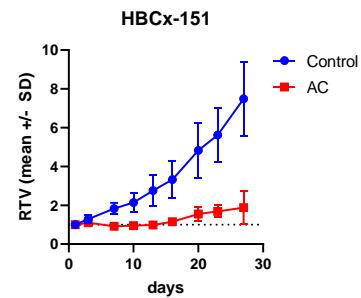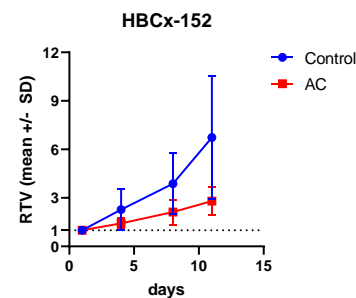

Supplement: Supplementary file 2 — Fig. S2. Response to AC chemotherapy in TNBC PDX (from HBCx‐31 to HBCx‐162). Adriamycin and cyclophosphamide were administered to the mice by the intraperitoneal (i.p.) route at the dose of 2 and 100mg/kg respectively, every 3 weeks Two cycles of AC treatment were administered and tumour response were evaluated at the end of the second cycle or when tumour volumes reached ethical sizes. Volumes are expressed relative to the initial volume, as a relative tumour volume (RTV). Mean +/‐SD, n=6‐8 mice/group. [file MOL2-17-2017-s005.pdf]

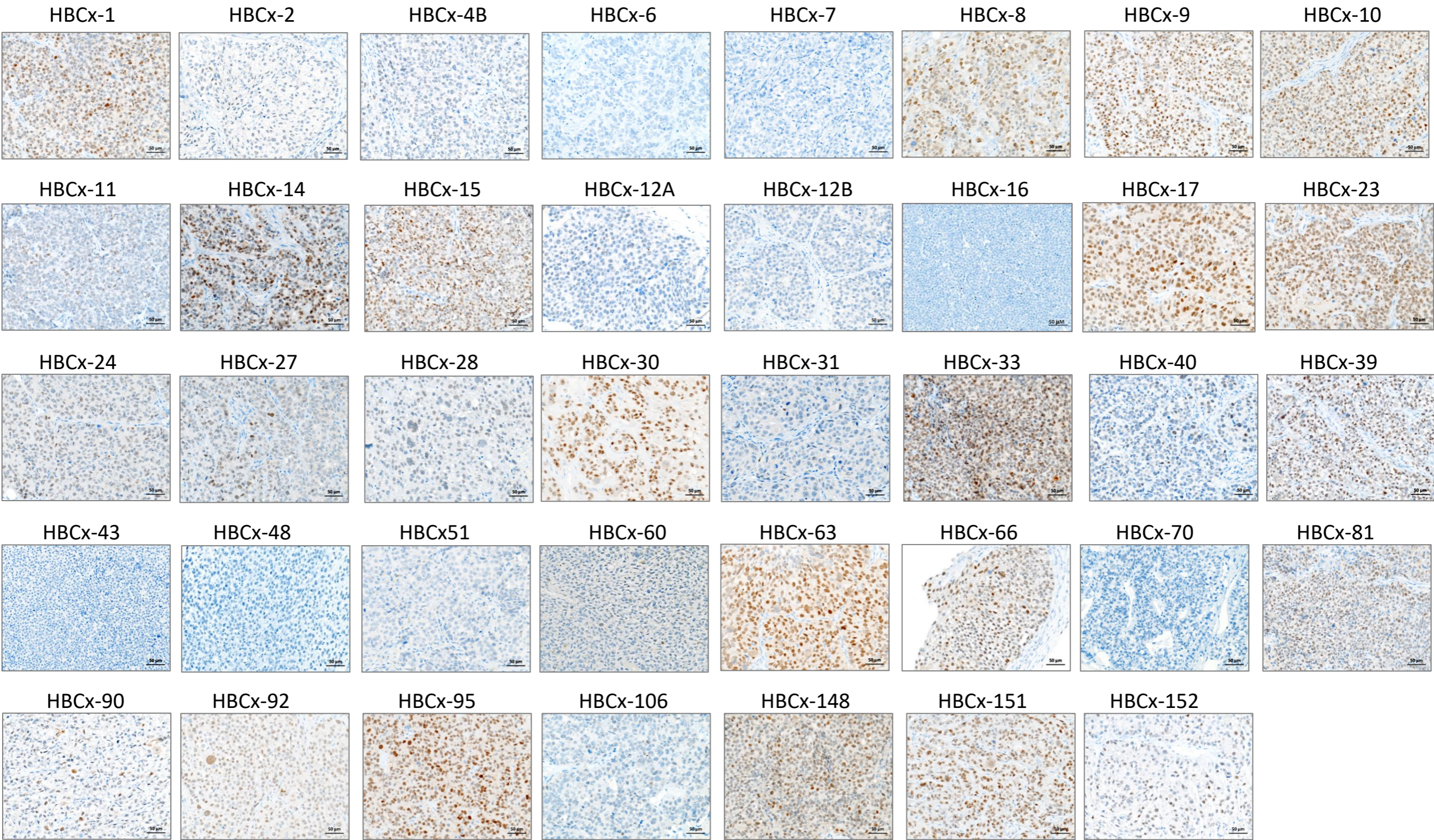

Supplement: Supplementary file 3 — Fig. S3. Immunohistochemistry analysis of HORMAD1 in the 39 PDX. Images are representative of the whole tissue sections; scale bar is 50 μm. [file MOL2-17-2017-s008.pdf]

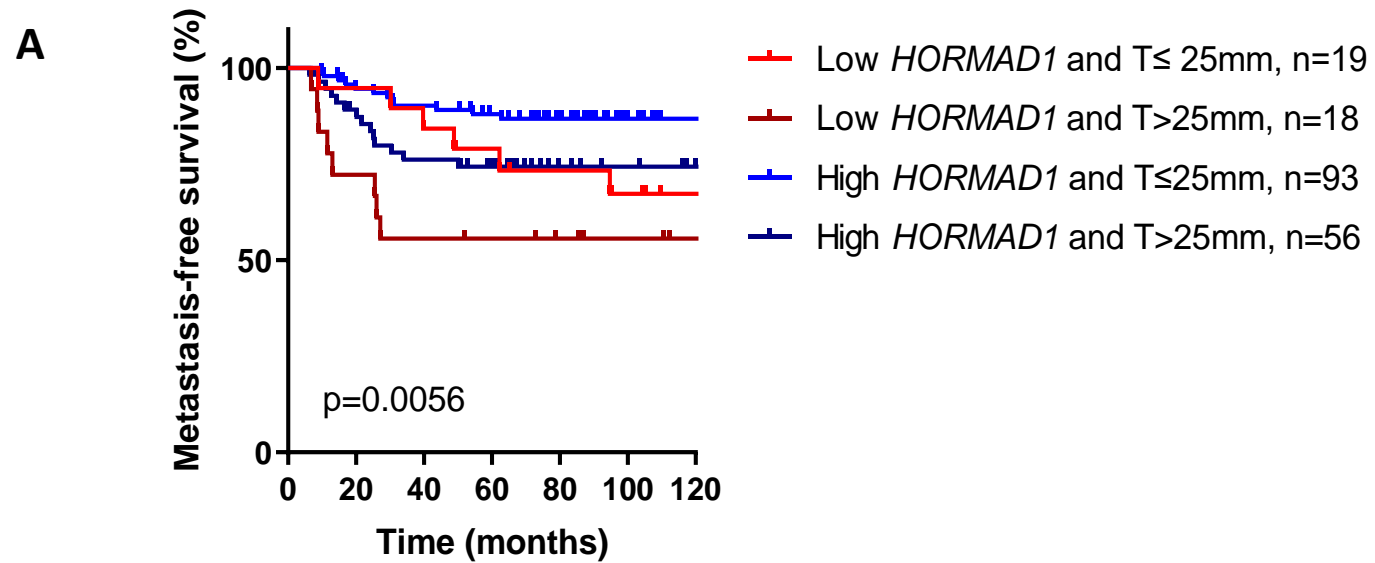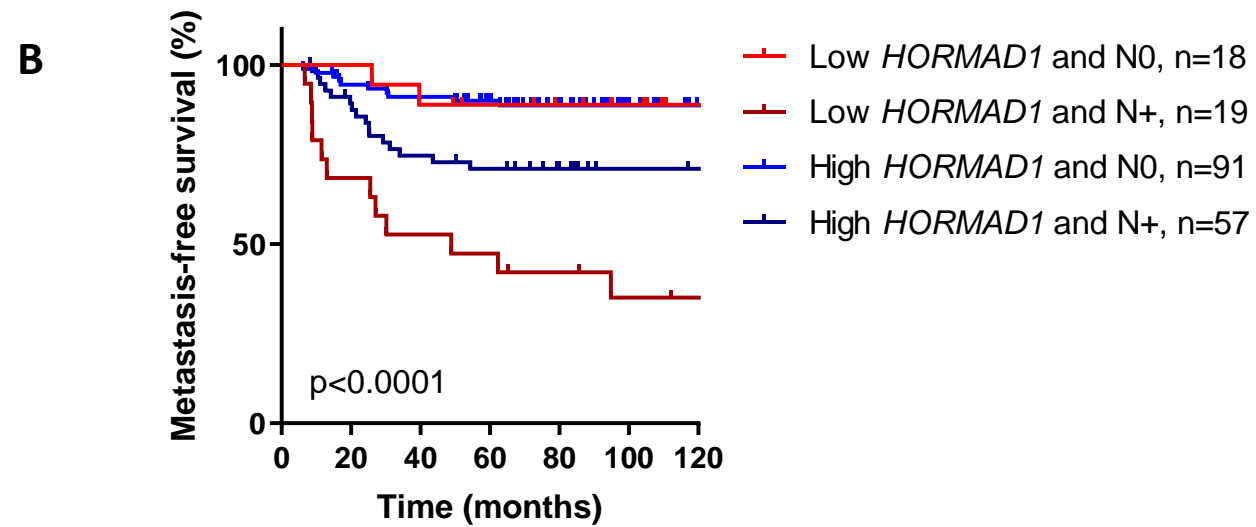

Supplement: Supplementary file 4 — Fig. S4. (A) MFS survival curves of TNBC patients stratified according to HORMAD1 status and macroscopic tumour size status (T). The patients with the poorest prognosis had low HORMAD1 expression and large tumour size (> 25 mn), while those with the best prognosis had high HORMAD1 expression and small tumour size (≤25mn). (B) MFS survival curves of TNBC patients stratified according to HORMAD1 status and LN status. The patients with the poorest prognosis had low HORMAD1 expression and positive LN while those with the best prognosis had negative LN (whatever the level of HORMAD1 expression). [file MOL2-17-2017-s006.pdf]
