# Supplementary material for: HORMAD1 overexpression predicts response to anthracycline–cyclophosphamide and survival in triple‐negative breast cancers
Source: Mol Oncol. 2023 Mar 23;17(10):2017–28. doi: 10.1002/1878-0261.13412 (PMC10552896; doi:10.1002/1878-0261.13412)
Supplement: Supplementary file 5 — Table S1. List of genes differentially expressed between PDXs treated by AC in the “response group (R)” as compared to “progressive disease (PD)” group. [file MOL2-17-2017-s009.docx]

**Table S1.** List of genes differentially expressed between PDXs treaeted by AC in the "response group (R)" as compared to "progressive disease (PD)" group.

| Transcript ID | Gene Symbol | RefSeq | p-value  (R vs. PD) | Fold-Change  (R vs. PD) | Fold-Change  (R vs. PD) (Description) |
| --- | --- | --- | --- | --- | --- |
| 8042788 | *ACTG2* | NM_001199893 | 0.01139830 | 4.06126 | R up vs PD |
| 8020724 | *DSG1* | NM_001942 | 0.0170301 | 3.4479 | R up vs PD |
| 7985159 | *CRABP1* | NM_004378 | 0.0154717 | 3.20418 | R up vs PD |
| 7919787 | *HORMAD1* | NM_001199829 | 0.0187439 | 3.17119 | R up vs PD |
| 8124531 | *HIST1H3I* | NM_003533 | 0.038282 | 3.10674 | R up vs PD |
| 8083233 | *ZIC1* | NM_003412 | 0.00029722 | 2.82673 | R up vs PD |
| 7964722 | *WIF1* | NM_007191 | 0.0223075 | 2.56112 | R up vs PD |
| 8031157 | *TTYH1* | NM_001005367 | 0.0204119 | 2.53889 | R up vs PD |
| 8117422 | *HIST1H4F* | NM_003540 | 0.00549146 | 2.49139 | R up vs PD |
| 8132118 | *AQP1* | NM_001185060 | 0.034137 | 2.46819 | R up vs PD |
| 7960919 | *MFAP5* | NM_001297709 | 0.0298465 | 2.46081 | R up vs PD |
| 8124534 | *HIST1H4L* | NM_003546 | 0.00085078 | 2.42921 | R up vs PD |
| 8124380 | *HIST1H1A* | NM_005325 | 0.00817645 | 2.42745 | R up vs PD |
| 8129562 | *CTGF* | NM_001901 | 0.00825624 | 2.4054 | R up vs PD |
| 8123876 | *LINC00518* | NR_027793 | 0.00154016 | 2.38326 | R up vs PD |
| 8057506 | *FRZB* | NM_001463 | 0.0452468 | 2.38056 | R up vs PD |
| 8113800 | *FBN2* | NM_001999 | 0.0202873 | 2.36785 | R up vs PD |
| 8077970 | *FBLN2* | NM_001004019 | 0.00524485 | 2.36262 | R up vs PD |
| 7909730 | *KCNK2* | NM_001017424 | 0.00346343 | 2.3407 | R up vs PD |
| 8121784 | *FABP7* | NM_001446 | 0.0122304 | 2.33093 | R up vs PD |
| 7968678 | *FREM2* | NM_207361 | 0.00775546 | 2.32204 | R up vs PD |
| 8016094 | *GJC1* | NM_001080383 | 0.0114457 | 2.29254 | R up vs PD |
| 8120719 | *CD109* | NM_001159587 | 0.0159958 | 2.23709 | R up vs PD |
| 8124166 | *MBOAT1* | NM_001080480 | 0.00671278 | 2.1823 | R up vs PD |
| 8174648 | *CT83* | NM_001017978 | 0.038758 | 2.16571 | R up vs PD |
| 7944082 | *TAGLN* | NM_001001522 | 0.0310138 | 2.12038 | R up vs PD |
| 8081219 | *ST3GAL6* | NM_001271142 | 0.0106338 | 2.11678 | R up vs PD |
| 8084524 | *EPHB3* | NM_004443 | 0.015282 | 2.11549 | R up vs PD |
| 8088560 | *ADAMTS9* | NM_182920 | 0.0458909 | 2.11279 | R up vs PD |
| 8014233 | *SLFN11* | NM_001104587 | 0.0409117 | 2.09971 | R up vs PD |
| 8175234 | *GPC3* | NM_001164617 | 0.0434861 | 2.09636 | R up vs PD |
| 8129573 | *MOXD1* | NM_015529 | 0.0279353 | 2.0956 | R up vs PD |
| 8081657 | *CD200* | NM_001004196 | 0.00255379 | 2.09336 | R up vs PD |
| 8172022 | *TMEM47* | NM_031442 | 0.0384856 | 2.08328 | R up vs PD |
| 8047926 | *MAP2* | NM_001039538 | 0.0261589 | 2.04564 | R up vs PD |
| 8085431 | *NUP210* | NM_024923 | 0.0070842 | 2.0427 | R up vs PD |
| 8094778 | *UCHL1* | NM_004181 | 0.0441392 | 2.03159 | R up vs PD |
| 8136347 | *CALD1* | NM_004342 | 0.0154411 | 2.02363 | R up vs PD |
| 8097017 | *UGT8* | NM_001128174 | 0.0431649 | 1.98874 | R up vs PD |
| 8078619 | *ITGA9* | NM_002207 | 0.0403563 | 1.97857 | R up vs PD |
| 8118890 | *SCUBE3* | NM_001303136 | 0.041884 | 1.97016 | R up vs PD |
| 7932584 | *PRTFDC1* | NM_001282786 | 0.00949279 | 1.967 | R up vs PD |
| 8117034 | *GMPR* | NM_006877 | 0.0147079 | 1.96262 | R up vs PD |
| 7986385 | *SYNM* | NM_015286 | 0.0439704 | 1.96141 | R up vs PD |
| 8124527 | *HIST1H1B* | NM_005322 | 0.0304146 | 1.95666 | R up vs PD |
| 8035201 | *CPAMD8* | NM_015692 | 0.00025019 | 1.95183 | R up vs PD |
| 8070632 | *CBS* | NM_000071 | 0.00919152 | 1.94279 | R up vs PD |
| 8138888 | *PDE1C* | NM_001191056 | 0.019691 | 1.94115 | R up vs PD |
| 8131666 | *ITGB8* | NM_002214 | 0.0474236 | 1.94063 | R up vs PD |
| 8123920 | *ELOVL2* | NM_017770 | 0.0157502 | 1.94032 | R up vs PD |
| 8167673 | *MAGED4* | NM_001098800 | 0.0156742 | 1.93739 | R up vs PD |
| 8172722 | *MAGED4* | NM_001098800 | 0.0156742 | 1.93739 | R up vs PD |
| 7954631 | *FAR2* | NM_001271783 | 0.035339 | 1.9323 | R up vs PD |
| 7905329 | *MLLT11* | NM_006818 | 0.0250897 | 1.93076 | R up vs PD |
| 7991224 | *HAPLN3* | NM_001307952 | 0.0350841 | 1.92316 | R up vs PD |
| 8079131 | *FAM198A* | NM_001129908 | 0.005908 | 1.92092 | R up vs PD |
| 8028924 | *MIA* | NM_001202553 | 0.0485877 | 1.91975 | R up vs PD |
| 8127932 | *TBX18* | NM_001080508 | 0.0256584 | 1.90952 | R up vs PD |
| 7906307 | *KIRREL* | NM_001286349 | 0.0440328 | 1.90643 | R up vs PD |
| 8148435 | *WISP1* | NM_001204869 | 0.0302614 | 1.8813 | R up vs PD |
| 8019842 | *TYMS* | NM_001071 | 0.00043277 | 1.87694 | R up vs PD |
| 7961524 | *ERP27* | NM_001300784 | 0.0376743 | 1.86273 | R up vs PD |
| 7918857 | *TSPAN2* | NM_001308315 | 0.00393928 | 1.85691 | R up vs PD |
| 8039044 | *ZNF415* | NM_001136038 | 0.00393746 | 1.85631 | R up vs PD |
| 8088680 | *EOGT* | NM_001278689 | 0.00200483 | 1.85323 | R up vs PD |
| 8119466 | *MDFI* | NM_001300804 | 0.0199865 | 1.83612 | R up vs PD |
| 8075992 | *SOX10* | NM_006941 | 0.0321439 | 1.83503 | R up vs PD |
| 7903507 | *FAM102B* | NM_001010883 | 0.0038685 | 1.83162 | R up vs PD |
| 7925250 | *GNG4* | NM_001098721 | 0.00569216 | 1.83076 | R up vs PD |
| 7972713 | *EFNB2* | NM_004093 | 0.0314289 | 1.8246 | R up vs PD |
| 8135224 | *NFE4* | NM_001085386 | 0.0123372 | 1.81696 | R up vs PD |
| 8107798 | *SLC27A6* | NM_001017372 | 0.012766 | 1.8145 | R up vs PD |
| 7972557 | *GPR183* | NM_004951 | 0.011099 | 1.81328 | R up vs PD |
| 8090098 | *MYLK* | NM_053025 | 0.0162603 | 1.80627 | R up vs PD |
| 8002882 | *CHST6* | NM_021615 | 0.0103957 | 1.78314 | R up vs PD |
| 7923762 | *KLHDC8A* | NM_001271863 | 0.0159976 | 1.77799 | R up vs PD |
| 7928959 | *PTEN* | NM_000314 | 0.0352257 | 1.76973 | R up vs PD |
| 8124521 | *HIST1H4K* | NM_003541 | 0.021579 | 1.76562 | R up vs PD |
| 8104314 | *IRX1* | NM_024337 | 0.0150324 | 1.76359 | R up vs PD |
| 7919560 | *RNVU1-15* | NR_104076 | 0.0134627 | 1.76176 | R up vs PD |
| 7957452 | *ALX1* | NM_006982 | 0.0301495 | 1.75726 | R up vs PD |
| 7941587 | *CNIH2* | NM_182553 | 0.0049209 | 1.75226 | R up vs PD |
| 8053417 | *CAPG* | NM_001256139 | 0.00662887 | 1.74959 | R up vs PD |
| 7995825 | *MT1F* | NM_001301272 | 0.0188736 | 1.74832 | R up vs PD |
| 8091757 | *TRIM59* | NM_173084 | 0.00204908 | 1.74478 | R up vs PD |
| 7919556 | *RNVU1-18* | NR_004400 | 0.0136707 | 1.74017 | R up vs PD |
| 8117120 | *ID4* | NM_001546 | 0.040676 | 1.7314 | R up vs PD |
| 7924309 | *ESRRG* | NM_001134285 | 0.00688095 | 1.73037 | R up vs PD |
| 8049239 | *C2orf82* | NM_206895 | 0.0220176 | 1.72498 | R up vs PD |
| 8149629 | *GFRA2* | NM_001165038 | 0.0184037 | 1.72438 | R up vs PD |
| 8155167 | *HRCT1* | NM_001039792 | 0.0209679 | 1.72297 | R up vs PD |
| 8115756 | *KCNMB1* | NM_004137 | 0.0399491 | 1.71825 | R up vs PD |
| 8072360 | *TCN2* | NM_000355 | 0.0234671 | 1.70557 | R up vs PD |
| 7940147 | *FAM111B* | NM_001142703 | 0.0460102 | 1.70222 | R up vs PD |
| 8124144 | *DEK* | NM_001134709 | 0.00041029 | 1.69209 | R up vs PD |
| 8092520 | *C3orf70* | NM_001025266 | 0.0052514 | 1.69175 | R up vs PD |
| 8088264 | *IL17RD* | NM_017563 | 0.0163003 | 1.69044 | R up vs PD |
| 8070182 | *RCAN1* | NM_001285389 | 0.00903136 | 1.6901 | R up vs PD |
| 8020164 | *GNAL* | NM_001142339 | 0.0386102 | 1.68923 | R up vs PD |
| 8103728 | *HMGB2* | NM_001130688 | 0.00213543 | 1.68775 | R up vs PD |
| 8077879 | *SYN2* | NM_003178 | 0.0047362 | 1.68704 | R up vs PD |
| 8179011 | *MOG* | NM_001008228 | 0.0259957 | 1.68566 | R up vs PD |
| 8174189 | *TMSB15A* | NM_021992 | 0.0100942 | 1.67956 | R up vs PD |
| 7969986 | *TNFSF13B* | NM_001145645 | 0.0342963 | 1.679 | R up vs PD |
| 8157804 | *OLFML2A* | NM_001282715 | 0.00447114 | 1.6705 | R up vs PD |
| 8044440 | *FBLN7* | NM_001128165 | 0.0391941 | 1.66341 | R up vs PD |
| 7999674 | *MYH11* | NM_001040113 | 0.0353507 | 1.66319 | R up vs PD |
| 8127031 | *MCM3* | NM_001270472 | 0.00023535 | 1.66256 | R up vs PD |
| 7924910 | *ACTA1* | NM_001100 | 0.00784779 | 1.65961 | R up vs PD |
| 8144758 | *ZDHHC2* | NM_016353 | 0.0425783 | 1.65915 | R up vs PD |
| 8156905 | *MSANTD3-TMEFF1* | NM_001198812 | 0.00748493 | 1.65824 | R up vs PD |
| 8025918 | *CNN1* | NM_001299 | 0.0228528 | 1.65467 | R up vs PD |
| 8163063 | *CTNNAL1* | NM_001286974 | 0.017189 | 1.65214 | R up vs PD |
| 8070129 | *DONSON* | NM_017613 | 9.46E-05 | 1.65076 | R up vs PD |
| 8149330 | *CTSB* | NM_001908 | 0.00674611 | 1.65002 | R up vs PD |
| 8171161 | *ARSE* | NM_000047 | 0.00358316 | 1.64227 | R up vs PD |
| 8176026 | *FLNA* | NM_001110556 | 0.0299576 | 1.6351 | R up vs PD |
| 8173059 | *WNK3* | NM_001002838 | 0.00923631 | 1.63155 | R up vs PD |
| 8046488 | *CDCA7* | NM_031942 | 0.0187964 | 1.62958 | R up vs PD |
| 7924760 | *ITPKB* | NM_002221 | 0.0168492 | 1.62865 | R up vs PD |
| 7970317 | *TFDP1* | NM_007111 | 0.00075409 | 1.62767 | R up vs PD |
| 7897378 | *PER3* | NM_001289861 | 0.0346362 | 1.62567 | R up vs PD |
| 7954055 | *APOLD1* | NM_001130415 | 0.00282857 | 1.62538 | R up vs PD |
| 7973745 | *FOXG1* | NM_005249 | 0.00175658 | 1.62467 | R up vs PD |
| 8007454 | *RND2* | NM_005440 | 0.00626464 | 1.62456 | R up vs PD |
| 8052654 | *PELI1* | NM_020651 | 0.0250288 | 1.62256 | R up vs PD |
| 7954511 | *STK38L* | NM_015000 | 0.0144521 | 1.617 | R up vs PD |
| 8021470 | *PMAIP1* | NM_021127 | 0.030403 | 1.61526 | R up vs PD |
| 8117598 | *HIST1H4J* | NM_021968 | 0.0175145 | 1.61494 | R up vs PD |
| 8086185 | *PLCD1* | NM_001130964 | 0.0298719 | 1.61393 | R up vs PD |
| 8100318 | *SGCB* | NM_000232 | 0.0289156 | 1.61302 | R up vs PD |
| 7969693 | *RAP2A* | NM_021033 | 0.00786165 | 1.61169 | R up vs PD |
| 7919715 | *ANP32E* | NM_001136478 | 0.0210783 | 1.61121 | R up vs PD |
| 8121632 | *KPNA5* | NM_002269 | 0.0139612 | 1.61089 | R up vs PD |
| 8141846 | *FBXL13* | NM_001111038 | 0.0142673 | 1.6091 | R up vs PD |
| 7924712 | *LIN9* | NM_001270409 | 0.00176145 | 1.60887 | R up vs PD |
| 7967993 | *FGF9* | NM_002010 | 0.0234144 | 1.6046 | R up vs PD |
| 8150138 | *TEX15* | NM_031271 | 0.0173051 | 1.60313 | R up vs PD |
| 7994769 | *CORO1A* | NM_001193333 | 0.0426997 | 1.60186 | R up vs PD |
| 8177709 | *MOG* | NM_001008228 | 0.0226012 | 1.60164 | R up vs PD |
| 7948249 | *SLC43A1* | NM_001198810 | 0.0160599 | 1.59712 | R up vs PD |
| 8151871 | *CCNE2* | NM_057749 | 0.031526 | 1.59347 | R up vs PD |
| 8085665 | *RFTN1* | NM_015150 | 0.0413927 | 1.59194 | R up vs PD |
| 8126770 | *CYP39A1* | NM_001278738 | 0.0255713 | 1.58813 | R up vs PD |
| 8118116 | *MICA* | NM_000247 | 0.0103554 | 1.58727 | R up vs PD |
| 8077370 | *SETMAR* | NM_001243723 | 0.00012965 | 1.58513 | R up vs PD |
| 8083677 | *SCHIP1* | NM_001197107 | 0.0407854 | 1.58447 | R up vs PD |
| 7972826 | *LINC00346* | NR_027701 | 0.0185377 | 1.58393 | R up vs PD |
| 7927288 | *FAM35BP* | NR_027632 | 0.0123599 | 1.58359 | R up vs PD |
| 8020100 | *RALBP1* | NM_006788 | 0.00178666 | 1.58012 | R up vs PD |
| 8160521 | *MOB3B* | NM_024761 | 0.0191511 | 1.57994 | R up vs PD |
| 7909568 | *DTL* | NM_001286229 | 0.00361265 | 1.57075 | R up vs PD |
| 7927267 | *FAM35BP* | NR_027632 | 0.0119454 | 1.56789 | R up vs PD |
| 7903565 | *GPSM2* | NM_013296 | 0.00745232 | 1.56784 | R up vs PD |
| 8138527 | *STEAP1B* | NM_001164460 | 0.037281 | 1.56705 | R up vs PD |
| 7986329 | *NR2F2* | NM_001145155 | 0.00565 | 1.56658 | R up vs PD |
| 8076704 | *SMC1B* | NM_001291501 | 0.0382094 | 1.56279 | R up vs PD |
| 7953218 | *RAD51AP1* | NM_001130862 | 0.0222864 | 1.56275 | R up vs PD |
| 8019857 | *NDC80* | NM_006101 | 0.0125969 | 1.5617 | R up vs PD |
| 8020254 | *SEH1L* | NM_001013437 | 0.00674679 | 1.5606 | R up vs PD |
| 7903162 | *TMEM56* | NM_001199679 | 0.0183885 | 1.55772 | R up vs PD |
| 8170602 | *ZNF185* | NM_001178106 | 0.0161427 | 1.55739 | R up vs PD |
| 8096808 | *CCDC109B* | NM_017918 | 0.0377208 | 1.55708 | R up vs PD |
| 7986350 | *ARRDC4* | NM_183376 | 0.0452502 | 1.55601 | R up vs PD |
| 7924603 | *LBR* | NM_002296 | 0.00339679 | 1.55378 | R up vs PD |
| 8058695 | *BARD1* | NM_000465 | 0.00302582 | 1.55323 | R up vs PD |
| 8092000 | *TERC* | NR_001566 | 0.0242189 | 1.55219 | R up vs PD |
| 8077944 | *CAND2* | NM_001162499 | 0.00084654 | 1.55105 | R up vs PD |
| 8073015 | *KDELR3* | NM_006855 | 0.0405648 | 1.55094 | R up vs PD |
| 7928909 | *FAM35A* | NM_019054 | 0.0343798 | 1.54927 | R up vs PD |
| 8077728 | *EMC3-AS1* | NR_103821 | 0.00293866 | 1.54869 | R up vs PD |
| 8141459 | *GAL3ST4* | NM_024637 | 0.0201572 | 1.54845 | R up vs PD |
| 8162601 | *ZNF367* | NM_153695 | 0.00267065 | 1.54754 | R up vs PD |
| 8157092 | *TMEM38B* | NM_018112 | 0.010082 | 1.54613 | R up vs PD |
| 7898967 | *NCMAP* | NM_001010980 | 0.0473744 | 1.54603 | R up vs PD |
| 8028194 | *ZNF382* | NM_001256838 | 0.0461531 | 1.54469 | R up vs PD |
| 8028084 | *APLP1* | NM_001024807 | 0.0227492 | 1.54239 | R up vs PD |
| 7940508 | *DAGLA* | NM_006133 | 0.00497858 | 1.54151 | R up vs PD |
| 8025672 | *SLC44A2* | NM_001145056 | 0.00874947 | 1.54136 | R up vs PD |
| 7987315 | *ACTC1* | NM_005159 | 0.00662582 | 1.53977 | R up vs PD |
| 8139270 | *RASA4CP* | NR_024116 | 0.00493023 | 1.5397 | R up vs PD |
| 7972840 | *TUBGCP3* | NM_001286277 | 0.00549528 | 1.53812 | R up vs PD |
| 8127346 | *RAB23* | NM_001278666 | 0.00591681 | 1.53753 | R up vs PD |
| 8053648 | *KRCC1* | NM_001304526 | 0.00194304 | 1.53293 | R up vs PD |
| 7991406 | *PRC1* | NM_001267580 | 0.00604311 | 1.53095 | R up vs PD |
| 8121895 | *TRMT11* | NM_001031712 | 0.0113278 | 1.52949 | R up vs PD |
| 7951807 | *CADM1* | NM_001098517 | 0.0335002 | 1.5294 | R up vs PD |
| 8129231 | *FAM184A* | NM_001100411 | 0.00222924 | 1.5277 | R up vs PD |
| 7918813 | *CSDE1* | NM_001007553 | 0.025934 | 1.52713 | R up vs PD |
| 8145418 | *CDCA2* | NM_152562 | 0.018838 | 1.52646 | R up vs PD |
| 8039054 | *ZNF347* | NM_001172674 | 0.00590163 | 1.52609 | R up vs PD |
| 8124524 | *HIST1H2AK* | NM_003510 | 0.0266041 | 1.52572 | R up vs PD |
| 8119858 | *POLH* | NM_001291969 | 0.00870158 | 1.52486 | R up vs PD |
| 8125919 | *FKBP5* | NM_001145775 | 0.0284906 | 1.52309 | R up vs PD |
| 8121768 | *PKIB* | NM_001270393 | 0.0149918 | 1.52151 | R up vs PD |
| 7999319 | *CARHSP1* | NM_001042476 | 0.00741108 | 1.51891 | R up vs PD |
| 8119161 | *PIM1* | NM_001243186 | 0.0339635 | 1.516 | R up vs PD |
| 8020267 | *CEP192* | NM_032142 | 0.00674774 | 1.51562 | R up vs PD |
| 8128329 | *MMS22L* | NM_198468 | 0.0178722 | 1.51319 | R up vs PD |
| 8074780 | *YPEL1* | NM_013313 | 0.0279124 | 1.5129 | R up vs PD |
| 8121861 | *NCOA7* | NM_001122842 | 0.0210691 | 1.51248 | R up vs PD |
| 7917954 | *FRRS1* | NM_001013660 | 0.0366159 | 1.51041 | R up vs PD |
| 8144082 | *C7orf13* | NR_026865 | 0.0437662 | 1.50659 | R up vs PD |
| 8117748 | *MOG* | NM_001008228 | 0.0229518 | 1.50584 | R up vs PD |
| 8169022 | *WBP5* | NM_001006612 | 0.0491387 | 1.50568 | R up vs PD |
| 7965094 | *E2F7* | NM_203394 | 0.00880121 | 1.50544 | R up vs PD |
| 8110886 | *MED10* | NM_032286 | 0.013814 | 1.50441 | R up vs PD |
| 7969736 | *FARP1* | NM_001001715 | 0.0364939 | 1.5036 | R up vs PD |
| 8008922 | *PPM1D* | NM_003620 | 0.00521441 | 1.50269 | R up vs PD |
| 8031293 | *KIR2DL2* | NM_014219 | 0.00387155 | 1.5023 | R up vs PD |
| 7942964 | *TMEM135* | NM_001168724 | 0.0055682 | -1.50075 | R down vs PD |
| 8153175 | *TRAPPC9* | NM_001160372 | 0.00263909 | -1.50855 | R down vs PD |
| 8060949 | *ANKEF1* | NM_001303472 | 0.0489278 | -1.51213 | R down vs PD |
| 8166184 | *CA5B* | NM_007220 | 0.0217491 | -1.51214 | R down vs PD |
| 7988970 | *FAM214A* | NM_001286495 | 0.00885353 | -1.51693 | R down vs PD |
| 7963590 | *CSAD* | NM_001244705 | 0.00215171 | -1.51901 | R down vs PD |
| 8172266 | *MIR221* | NR_029635 | 0.0228339 | -1.52293 | R down vs PD |
| 7995421 | *LONP2* | NM_001300948 | 0.00173145 | -1.52302 | R down vs PD |
| 8015769 | *BRCA1* | NM_007294 | 0.0472848 | -1.52834 | R down vs PD |
| 7959312 | *TMEM120B* | NM_001080825 | 0.00585661 | -1.52846 | R down vs PD |
| 8151890 | *TP53INP1* | NM_001135733 | 0.0240167 | -1.52848 | R down vs PD |
| 7995539 | *NOD2* | NM_001293557 | 0.0212542 | -1.54091 | R down vs PD |
| 7970381 | *LOC102723462* | XR_951417 | 0.0336118 | -1.54766 | R down vs PD |
| 7951372 | *CASP4* | NM_001225 | 0.0488935 | -1.55381 | R down vs PD |
| 8066786 | *ZMYND8* | NM_001281769 | 0.00493445 | -1.55674 | R down vs PD |
| 7987369 | *DPH6* | NM_001141972 | 0.00220374 | -1.55805 | R down vs PD |
| 8148694 | *GRINA* | NM_000837 | 0.00188139 | -1.56137 | R down vs PD |
| 7987248 | *GOLGA8A* | NM_181077 | 0.0279919 | -1.56276 | R down vs PD |
| 7951397 | *CASP1* | NM_001223 | 0.0489119 | -1.56927 | R down vs PD |
| 8023497 | *ATP8B1* | NM_005603 | 0.0447632 | -1.57275 | R down vs PD |
| 8062658 | *LPIN3* | NM_001301860 | 0.00034208 | -1.57891 | R down vs PD |
| 7987279 | *GOLGA8B* | NM_001023567 | 0.0255517 | -1.58851 | R down vs PD |
| 7898516 | *ACTL8* | NM_030812 | 0.0349765 | -1.59525 | R down vs PD |
| 7995479 | *PAPD5* | NM_001040284 | 0.00127129 | -1.60727 | R down vs PD |
| 7985934 | *SEMA4B* | NM_020210 | 0.0302221 | -1.61828 | R down vs PD |
| 8009685 | *SLC9A3R1* | NM_004252 | 0.026791 | -1.61849 | R down vs PD |
| 8113103 | *KIAA0825* | NM_001145678 | 0.0418021 | -1.61953 | R down vs PD |
| 7979179 | *ERO1A* | NM_014584 | 0.0366244 | -1.61975 | R down vs PD |
| 8061497 | *FRG1BP* | NR_003579 | 0.00309683 | -1.62384 | R down vs PD |
| 8153426 | *MROH6* | NM_001100878 | 0.00408394 | -1.62796 | R down vs PD |
| 7897620 | *PGD* | NM_001304451 | 0.00465988 | -1.62935 | R down vs PD |
| 8148917 | *MFSD3* | NM_138431 | 0.00133598 | -1.63746 | R down vs PD |
| 7949971 | *CPT1A* | NM_001031847 | 0.0155334 | -1.64102 | R down vs PD |
| 7913667 | *GALE* | NM_000403 | 0.00119207 | -1.64212 | R down vs PD |
| 8133876 | *CD36* | NM_000072 | 0.0457394 | -1.64325 | R down vs PD |
| 8004510 | *CD68* | NM_001040059 | 0.017249 | -1.65873 | R down vs PD |
| 8146921 | *RDH10* | NM_172037 | 0.0406928 | -1.65983 | R down vs PD |
| 7974697 | *DAAM1* | NM_001270520 | 0.0047232 | -1.66976 | R down vs PD |
| 8063211 | *NCOA3* | NM_001174087 | 0.00525754 | -1.67187 | R down vs PD |
| 8164535 | *CRAT* | NM_000755 | 0.0452834 | -1.67256 | R down vs PD |
| 7957126 | *KCNMB4* | NM_014505 | 0.0252279 | -1.70006 | R down vs PD |
| 8062873 | *KCNK15* | NM_022358 | 0.00744794 | -1.70888 | R down vs PD |
| 8151952 | *NIPAL2* | NM_024759 | 0.0127926 | -1.72636 | R down vs PD |
| 8024062 | *CFD* | NM_001928 | 0.0133808 | -1.72658 | R down vs PD |
| 7988414 | *GATM* | NM_001482 | 0.0135399 | -1.75207 | R down vs PD |
| 8109629 | *FABP6* | NM_001040442 | 0.0110818 | -1.75281 | R down vs PD |
| 7956759 | *SRGAP1* | NM_020762 | 0.00679851 | -1.76836 | R down vs PD |
| 8032392 | *MKNK2* | NM_017572 | 0.00068538 | -1.7729 | R down vs PD |
| 8153424 | *MROH6* | NM_001100878 | 0.0130358 | -1.77548 | R down vs PD |
| 8153430 | *NAPRT* | NM_001286829 | 0.00157343 | -1.82226 | R down vs PD |
| 7993588 | *TMC7* | NM_001160364 | 0.00436309 | -1.8231 | R down vs PD |
| 7940775 | *RARRES3* | NM_004585 | 0.0185425 | -1.86394 | R down vs PD |
| 8108873 | *ARHGAP26* | NM_001135608 | 0.00017916 | -1.94068 | R down vs PD |
| 8013660 | *ALDOC* | NM_005165 | 0.0154894 | -1.96447 | R down vs PD |
| 7944667 | *SORL1* | NM_003105 | 0.028833 | -1.99462 | R down vs PD |
| 7901287 | *CYP4Z1* | NM_178134 | 0.0180641 | -2.27252 | R down vs PD |
| 7969003 | *ITM2B* | NM_021999 | 0.00318585 | -2.49375 | R down vs PD |
| 8026398 | *CASP14* | NM_012114 | 0.0371274 | -2.8102 | R down vs PD |
| 7983239 | *CKMT1B* | NM_020990 | 0.00052647 | -3.38964 | R down vs PD |
| 7983256 | *CKMT1B* | NM_020990 | 0.00052647 | -3.38964 | R down vs PD |
| 7969017 | *RB1* | NM_000321 | 0.00011876 | -3.51568 | R down vs PD |
