# Supplementary material for: HORMAD1 overexpression predicts response to anthracycline–cyclophosphamide and survival in triple‐negative breast cancers
Source: Mol Oncol. 2023 Mar 23;17(10):2017–28. doi: 10.1002/1878-0261.13412 (PMC10552896; doi:10.1002/1878-0261.13412)
Supplement: Supplementary file 6 — Table S2. statistical analysis of Fig. 1E (HORMAD1 expression in the different subgroups of TNBC). [file MOL2-17-2017-s001.docx]

**Table S2: statistical analysis of HORMAD1 gene expression in the different TNBC subtypes.**

| Number of families | 1 |  |  |  |  |
| --- | --- | --- | --- | --- | --- |
| Number of comparisons per family | 15 |  |  |  |  |
| Alpha | 0,05 |  |  |  |  |
|  |  |  |  |  |  |
| Uncorrected Fisher's LSD | Mean Diff, | 95,00% CI of diff, | Below threshold? | Summary | Individual P Value |
| BL1 vs. BL2 | 2,869 | 1,436 to 4,301 | Yes | *** | 0,0002 |
| BL1 vs. LAR | 3,567 | 1,932 to 5,201 | Yes | **** | <0,0001 |
| BL1 vs. M | 0,2752 | -0,8771 to 1,427 | No | ns | 0,6351 |
| BL1 vs. MSL | 2,889 | 1,018 to 4,760 | Yes | ** | 0,0030 |
| BL1 vs. IM | 1,273 | -0,4642 to 3,010 | No | ns | 0,1482 |
| BL2 vs. LAR | 0,6980 | -1,177 to 2,573 | No | ns | 0,4601 |
| BL2 vs. M | -2,593 | -4,067 to -1,119 | Yes | *** | 0,0008 |
| BL2 vs. MSL | 0,02080 | -2,064 to 2,105 | No | ns | 0,9842 |
| BL2 vs. IM | -1,596 | -3,561 to 0,3695 | No | ns | 0,1098 |
| LAR vs. M | -3,291 | -4,963 to -1,620 | Yes | *** | 0,0002 |
| LAR vs. MSL | -0,6772 | -2,906 to 1,551 | No | ns | 0,5461 |
| LAR vs. IM | -2,294 | -4,411 to -0,1765 | Yes | * | 0,0342 |
| M vs. MSL | 2,614 | 0,7113 to 4,517 | Yes | ** | 0,0078 |
| M vs. IM | 0,9976 | -0,7738 to 2,769 | No | ns | 0,2649 |
| MSL vs. IM | -1,617 | -3,921 to 0,6879 | No | ns | 0,1660 |
