# Supplementary material for: HORMAD1 overexpression predicts response to anthracycline–cyclophosphamide and survival in triple‐negative breast cancers
Source: Mol Oncol. 2023 Mar 23;17(10):2017–28. doi: 10.1002/1878-0261.13412 (PMC10552896; doi:10.1002/1878-0261.13412)
Supplement: Supplementary file 7 — Table S3. Histopathological and clinical characteristics of 526 breast cancer patients. aLog‐rank test (521 samples with MFS > 6 months). NS: not significant; bScarff Bloom Richardson classification, cInformation available for 511 patients; dInformation available for 521 patients; eInformation available for 516 patients. [file MOL2-17-2017-s007.docx]

**Table S3. Histopathological and clinical characteristics of 526 breast cancer patients**

|  | Number of patients (%) | Number with metastases (%) | p-valuea |
| --- | --- | --- | --- |
|  |  |  |  |
| *Total* | 526 (100) | 209 (39.7) |  |
|  |  |  |  |
| *Age*  ≤50  >50 | 125 (23.8)  401 (76.2) | 52 (41.6)  157 (39.2) | 0.71 (NS) |
| *SBR* ^b^ *histological grade* ^c^  I  II  III | 60 (11.7)  241 (47.2)  210 (41.1) | 12 (20.0)  100 (41.5)  93 (44.3) | **0.0012** |
| *Lymph node status* ^d^  0  1-3  >3 | 160 (30.7)  248 (47.6)  113 (21.7) | 48 (30.0)  87 (35.1)  72 (63.7) | **<0.0001** |
| *Macroscopic tumor size ^e^*  ≤25mm  >25mm | 248 (48.1)  268 (51.9) | 76 (30.6)  132 (49.3) | **<0.0001** |
| *ERα status*  Negative  Positive | 181 (34.4)  345 (65.6) | 76 (42.0)  133 (38.6) | 0.14 (NS) |
| *PR status*  Negative  Positive | 254 (48.3)  272 (51.7) | 109 (42.9)  100 (36.8) | **0.032** |
| *ERBB2 status*  Negative  Positive | 395 (75.1)  131 (24.9) | 152 (38.5)  57 (43.5) | 0.39 (NS) |
| *Molecular subtypes*  HR- ERBB2-  HR- ERBB2+  HR+ ERBB2-  HR+ ERBB2+ | 101 (19.2)  73 (13.9)  294 (55.9)  58 (11.0) | 38 (37.6)  36 (49.3)  114 (38.8)  21 (36.2) | 0.11 (NS) |
|  |  |  |  |

a Log-rank test (521 samples with MFS>6 months). NS: not significant

b Scarff Bloom Richardson classification

c Information available for 511 patients

d Information available for 521 patients

e Information available for 516 patients
