# Supplementary material for: HORMAD1 overexpression predicts response to anthracycline–cyclophosphamide and survival in triple‐negative breast cancers
Source: Mol Oncol. 2023 Mar 23;17(10):2017–28. doi: 10.1002/1878-0261.13412 (PMC10552896; doi:10.1002/1878-0261.13412)
Supplement: Supplementary file 8 — Table S4. Histopathological and clinical characteristics of 186 triple negative breast cancer patients. NS: not significant, AC: anthracyclines cyclophosphamide; aLog‐rank test, bScarff Bloom Richardson classification, cInformation available for 186 patients; dInformation available for 153 patients. [file MOL2-17-2017-s003.docx]

**Table S4. Histopathological and clinical characteristics of 186 triple negative breast cancer patients**

|  | Number of patients (%) | Number with metastases (%) | *p-*value MFS^a^ | Number of death (%) | *p-*value  OS^a^ |
| --- | --- | --- | --- | --- | --- |
| *Total* | 186 (100.0) | 43 (23.1) |  | 42 (22.6) |  |
| *Age*  ≤50  >50 | 78 (41.9)  108 (58.1) | 18 (23.1)  25 (23.1) | 0.84 (NS) | 18 (23.1)  24 (22.2) | 0.64 (NS) |
| *SBR* *histological grade* ^b^  II  III | 15 (8.1)  171 (91.9) | 2 (13.3)  41 (24.0) | 0.29 (NS) | 3 (20.0)  39 (22.8) | 0.77 (NS) |
| *Lymph node status* ^c^  negative  positive | 109 (58.9)  76 (41.1) | 12 (11.0)  31 (40.8) | **<0.0001** | 9 (8.3)  33 (43.4) | **<0.0001** |
| *Macroscopic tumor size*  ≤25mm  >25mm | 112 (60.2)  74 (39.8) | 19 (17.0)  24 (32.4) | **0.0090** | 19 (17.0)  23 (31.1) | **0.011** |
| *Radiotherapy* ^c^  Yes  No | 168 (90.8)  17 (9.2) | 39 (23.2)  4 (23.5) | 0.82 (NS) | 39 (23.2)  3 (17.6) | 0.98 (NS) |
| *Chemotherapy regimen* ^d^  AC  Anthracyclines alone  other  no chemotherapy | 122 (79.7)  19 (12.4) 10 (6.5)  2 (1.3) | 23 (18.9)  5 (26.3)  4 (40.0)  0 (0.0) | 0.19 (NS) | 25 (20.5)  3 (15.8)  4 (40.0)  0 (0.0) | 0.23 (NS) |

NS: not significant, AC: anthracyclines cyclophosphamide; a Log-rank test, b Scarff Bloom Richardson classification, c Information available for 186 patients; d Information available for 153 patients.
