# Supplementary material for: HORMAD1 overexpression predicts response to anthracycline–cyclophosphamide and survival in triple‐negative breast cancers
Source: Mol Oncol. 2023 Mar 23;17(10):2017–28. doi: 10.1002/1878-0261.13412 (PMC10552896; doi:10.1002/1878-0261.13412)
Supplement: Supplementary file 9 — Table S5. Multivariate COX analysis of MFS for HORMAD1 in the series of 186 triple negative breast cancers. aHazard ratio. b95% Confidence Interval. cMultivariate COX analysis. [file MOL2-17-2017-s004.docx]

**Table S5: Multivariate COX analysis of MFS for *HORMAD1* in the series of 186 triple negative breast cancers**

| Characteristics | | HR ^a^ | 95% CI ^b^ | *p*-value ^c^ |
| --- | --- | --- | --- | --- |
|  |  |  |  |  |
| *HORMAD1* | low | 1.0 |  | **0.028** |
|  | high | 0.49 | 0.26-0.93 |  |
|  |  |  |  |  |
| *Macroscopic tumor size* | ≤25mm | 1.0 |  | **0.029** |
|  | >25mm | 1.96 | 1.07-3.59 |  |
|  |  |  |  |  |
| *Lymph node status* | negative | 1.0 |  | **0.00011** |
|  | positive | 3.78 | 1.93-7.41 |  |

^a^ Hazard ratio. ^b^ 95% Confidence Interval. ^c^ Multivariate COX analysis.
